# Hydrogen Bonding and Noncovalent Electric Field Effects in the Photoconversion of a Phytochrome

**DOI:** 10.1021/acs.jpcb.4c06419

**Published:** 2024-11-19

**Authors:** Anh Duc Nguyen, Norbert Michael, Luisa Sauthof, Johannes von Sass, Oanh Tu Hoang, Andrea Schmidt, Mariafrancesca La Greca, Ramona Schlesinger, Nediljko Budisa, Patrick Scheerer, Maria Andrea Mroginski, Anastasia Kraskov, Peter Hildebrandt

**Affiliations:** a Institut für Chemie, Sekr. C7, Technische Universität Berlin, Straße des 17. Juni 115, Berlin D-10623, Germany; b Institut für Chemie, Sekr. PC14, Technische Universität Berlin, Straße des 17. Juni 135, Berlin D-10623, Germany; c Institute of Medical Physics and Biophysics, Group Structural Biology of Cellular Signaling, Charité − Universitätsmedizin Berlin, Corporate member of Freie Universität Berlin and Humboldt-Universität zu Berlin, Charitéplatz 1, Berlin D-10117, Germany; d Experimental Physics: Genetic Biophysics, Freie Universität Berlin, Arnimallee 14, Berlin D-14195, Germany; e Department of Chemistry, University of Manitoba, 144 Dysart Road, Winnipeg, Manitoba R3T 2N2, Canada

## Abstract

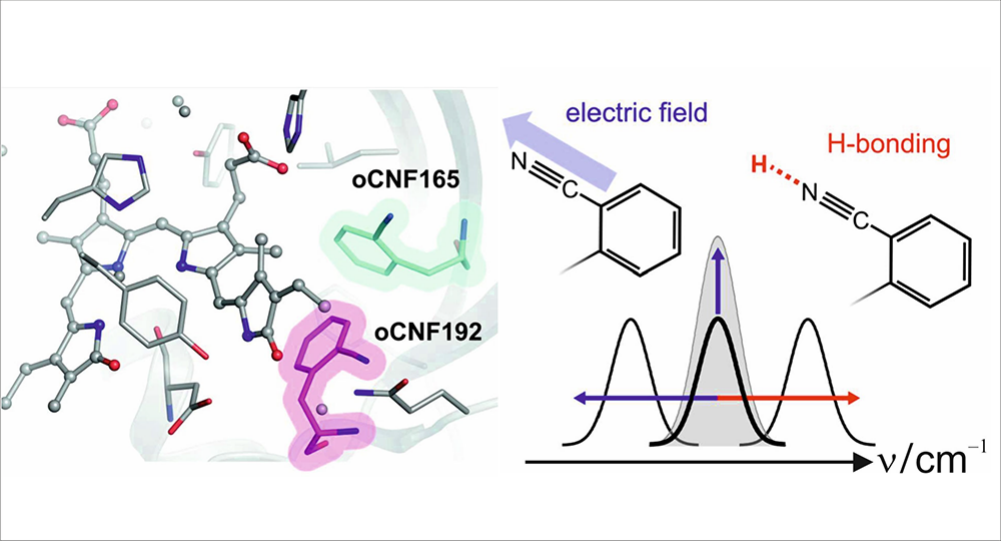

A profound understanding of protein structure and mechanism requires dedicated experimental and theoretical tools to elucidate electrostatic and hydrogen bonding interactions in proteins. In this work, we employed an approach to disentangle noncovalent and hydrogen-bonding electric field changes during the reaction cascade of a multidomain protein, i.e., the phytochrome Agp2. The approach exploits the spectroscopic properties of nitrile probes commonly used as reporter groups of the vibrational Stark effect. These probes were introduced into the protein through site-specific incorporation of noncanonical amino acids resulting in four variants with different positions and orientations of the nitrile groups. All substitutions left structures and the reaction mechanism unchanged. Structural models of the dark states (Pfr) were used to evaluate the total electric field at the nitrile label and its transition dipole moment. These quantities served as an internal standard to calculate the respective properties of the photoinduced products (Lumi-F, Meta-F, and Pr) based on the relative intensities of the nitrile stretching bands. In most cases, the spectral analysis revealed two substates with a nitrile in a hydrogen-bonded or hydrophobic environment. Using frequencies and intensities, we managed to extract the noncovalent contribution of the electric field from the individual substates. This analysis resulted in profiles of the noncovalent and hydrogen-bond-related electric fields during the photoinduced reaction cascade of Agp2. These profiles, which vary significantly among the four variants due to the different positions and orientations of the nitrile probes, were discussed in the context of the molecular events along the Pfr → Pr reaction cascade.

## Introduction

Biochemical processes of proteins are typically initiated by external stimuli, such as binding of ligands, electron transfer, or photoexcitation. These signals are subsequently translated into protein structural changes, which may cover various time and length scales and eventually induce biological functions. In most cases, details of the underlying molecular “causal chain” are enigmatic, but it is quite likely that different kinds of intramolecular forces are involved.^1−3^ Among them, electrostatics and their spatiotemporal changes probably play an important role as already proposed 50 years ago.^1^

However, for a long time, such electrostatic interactions, which are associated with the translocation of charges, reorientations of amino acid side chains and water molecules, and fluctuations of dipoles, could hardly be determined experimentally. Hence, this concept was mainly supported by theoretical predictions.^1,3−5^ The situation has started to change in the past 20 years with the pioneering work of Boxer’s group who adapted the vibrational Stark effect (VSE) to study local electrostatics in proteins.^6^ The VSE is based on the electric field dependence of the frequencies of localized vibrational modes such as the stretching modes of carbonyl and nitrile groups. The VSE approach further profited from developments in protein modification and engineering that allowed the site-directed incorporation of Stark effect reporter groups into the protein.^7^ A particularly interesting Stark effect reporter is the nitrile group because its stretching mode gives rise to an IR band in a region free of any protein bands.^6−13^ However, the frequency of this mode is sensitive to both noncovalent electric field and hydrogen bonding (H-bonding) interactions, which cause frequency shifts in opposite directions.^14−16^ Hence, frequency shifts of nitrile groups in H-bonding environments can hardly be analyzed in terms of local electric fields. Conversely, the transition dipole moment (TDM) of the vibrational transition, which is proportional to the square root of the other spectroscopic observable, the band intensity, also depends on the electric field but is independent of H-bonding interactions, as was recently demonstrated by Boxer and co-workers.^16^ For a molecule at a known concentration, the TDM can readily be determined on the basis of integral extinction of the measured band envelope. This approach has been successfully applied to the labeled photoactive yellow protein (PYP). A serious drawback of this approach, however, refers to proteins larger than PYP (14 kDa), which cannot be prepared in solutions of sufficiently high concentrations. In these cases, IR spectra are measured from protein films, for which the effective concentration is difficult to determine.

A way to circumvent this difficulty is the introduction of an internal standard, to which all of the observed changes can be related. This is also advantageous for studying changes in the electric field during a reaction sequence of a protein. Here, we have developed such an approach for the 65 kDa photosensory core module of a phytochrome, a red-light photosensor found in plants, bacteria, and fungi.^17^ Phytochromes harbor a methine-bridged tetrapyrrole chromophore as a light-sensing unit, which upon light absorption undergoes a photoisomerization at the terminal methine bridge followed by a cascade of structural relaxations (Supporting Information, Figure S1).^18−21^ This reaction sequence interconverts the parent states Pr and Pfr and involves proton translocation, which has been shown to be a prerequisite for the functional secondary structure transition of the tongue, a phytochrome-specific peptide segment. In view of these and other findings,^22−25^ it was proposed that changes of the electrostatics in the chromophore binding pocket (CBP) play a key role for transmitting photoisomerization of the chromophore to structural changes in the protein, which in turn affect the activity status of the enzymatic output module.^20,22,23^

In our previous work, we studied the electrostatics in the CBP of the bathy phytochrome Agp2 during the photoconversion from the dark-adapted Pfr state to the light-activated Pr state.^23^ Agp2 originates from *Agrobacterium fabrum* and carries a biliverdin (BV) chromophore. We introduced a noncanonical amino acid, *para*-cyanophenylalanine (pCNF), at two positions close to the chromophore, i.e., Tyr165 and Phe192. The resultant Agp2 variants Y165pCNF and F192pCNF remained structurally and functionally intact. In particular, proton transfer and restructuring of the tongue were fully preserved. The local electric fields at the nitrile probes were analyzed based on the frequency shifts of the CN stretching mode. For both variants, the largest shifts were observed upon the decay to Pr in the last step of the Pfr → Pr photocycle. However, reliable quantification of the electric fields was not possible due to the interference from H-bonding interactions.

In the present work, we have generated two further variants with *ortho*-cyanophenylalanine (oCNF) at positions 165 or 192. Similar to pCNF, the substitution by oCNF was found to be largely noninvasive as demonstrated by resonance Raman (RR) and IR difference spectroscopy. Thus, Y165oCNF and F192oCNF, along with the previously characterized variants Y165pCNF and F192pCNF, formed the basis for analyzing local electric field changes during the Pfr → Pr photoconversion using both the nitrile stretching mode intensities and frequencies. The novelty of our methodological approach is the definition and use of an internal standard that *(i)* circumvents tedious determination of the protein concentrations; *(ii)* allows extending the analysis to instable, here cryo-trapped, states; and *(iii)* most importantly, constitutes the pillar for linking spectroscopic observables with calculated properties of the Stark probe obtained by quantum mechanical/molecular mechanics (QM/MM) and molecular dynamics (MD) methods. The internal standards are provided by the Pfr states of the four variants for which either the crystal structures were solved or very good structural models were available. The approach provides a consistent analysis of the total electric fields in the various states of the photoreceptor, including all spectroscopically distinguishable subpopulations. In addition, we have succeeded in sorting out noncovalent and H-bonding contributions to the electric field, thereby providing a deeper insight into the relevance of local electrostatic fields for inducing relevant structural changes in proteins.

## Materials and Methods

### Protein Expression

The variants F192oCNF and Y165oCNF of the Agp2 photosensory core module (Agp2-PCM) including oCNF Stark labels were produced in a similar way as described previously.^23^ We used the N346A/C348A mutated pyrrolysyl-tRNA synthetase (PylRS), which was developed by the Liu group for genetic incorporation of a variety of noncanonical amino acids (NAAs).^26^ The expression system followed the scheme T7Promotor–RBS–*Agp2PCM*–RBS–mPylRS–T7Terminator–ProcABKPromotor–pyltRNA–ProcTerminator. The DNA sequence of the *Agp2PCM* gene (PAS-GAF-PHY; NCBI GenBank ID AAK87910) was the same as described in Schmidt et al.^27^ The AA sequence of *Methanosarcina mazei* pyrrolysyl-tRNA synthetase (mmPylRS) was taken from UNIPROT (Q8PWY1). The sequence was provided with the N346A/C348A mutations and *E. coli* codon-optimized back-translated to DNA.^28−30^ The DNA sequence for the ProcABKPromotor–pyltRNA–ProcTerminator cassette was taken from the plasmid pEVOL-pylT-N346A/C348A without alteration.^26^ The DNA RBS–mmPylRS–T7Terminator–ProcABKPromotor–pyltRNA–ProcTerminator was synthesized by Eurofins and ligated in a pET21b Agp2PCM-6His expression plasmid from ref (27). Finally, the *Agp2PCM* gene was mutated with the nonsense codon TAG at the desired position for oCNF incorporation. The plasmids were transformed into *E. coli* BL21-DE3. The *E. coli* cells were grown in M9–glucose–ampicillin medium at 30 °C to a cell density of OD 0.6 at 600 nm and then cooled on ice to 5 °C. oCNF (Santa Cruz Biotech) and IPTG (Sigma) were added as solids to concentrations of 1.3 mM and 90 μM, respectively. After dissolution, the cells were warmed and grown overnight at 20 °C. Subsequently, the cells were disrupted with a French pressure cell. Soluble proteins were subjected to affinity chromatography with Protino-NiNTA Agarose (Macherey-Nagel) to isolate the His-tagged apo-phytochrome. Biliverdin (Frontier Scientific) was added at an ∼3 molar excess to the apo-phytochrome. The resultant holophytochrome was concentrated by ammonium sulfate precipitation and finally purified by size exclusion chromatography (Sephacryl300HR, GE Healthcare). The expression of the F192pCNF and Y165pCNF was described previously.^23^

### Crystallization and Structure Determination

The Agp2 variants F192oCNF and Y165oCNF were methylated and crystals were grown according to our previously published conditions.^27^ To improve the quality of the crystals, a seeding procedure was introduced in an analogue crystallization setup. The highest resolution diffraction data for F192oCNF and Y165oCNF were collected at the ESRF synchrotron (Grenoble, France) at beamline ID30B^31^ and ID23-2^32^ using a Pilatus3 6M and Pilatus3 2M detector with a wavelength of λ = 0.97625 and 0.87317 Å, respectively. All images were indexed, integrated, and scaled using the XDS program package^33^ and the CCP4^34^ programs SCALA^35^ and AIMLESS.^36^ Crystals of both variants belong to the orthorhombic space group *P*2_1_2_1_2_1_ (cell parameters for F192oCNF: *a* = 74.6 Å, *b* = 93.6 Å, *c* = 174.0 Å, α = β = γ = 90°; cell parameters for Y165oCNF: *a* = 74.7 Å, *b* = 93.8 Å, *c* = 174.6 Å, α = β = γ = 90°). Table S1 summarizes the statistics for the crystallographic data collection and structural refinement. The structure determination was performed as described previously.^27^ Here, wild-type (WT) Agp2-PCM (PDB entry 6G1Y) was used as the initial search model for initial phases obtained with PHASER^37^ by molecular replacement (rotation, translation, rigid-body fitting). Simulated annealing with the resulting models was performed using a slow-cooling protocol and maximum likelihood target function, energy minimization, and B-factor refinement by the program PHENIX.^38^ All crystallographic structures were modeled with TLS refinement^39^ using anisotropic temperature factors for all protein atoms. Restrained, individual B-factors were refined, and the crystal structures were finalized by the CCP4 program REFMAC5^40^ and other programs of the CCP4 suite.^34^ The agreement factors *R*_free_ and *R*_cryst_ of the final models are 22.4 and 18.6% for F192oCNF and 22.3 and 18.5% for Y165oCNF, respectively (Table S1). Manual rebuilding of the crystal structure models and electron density interpretation were performed after each refinement step using the program COOT.^41^ All molecular graphic representations in this work were created using PyMOL.^42^

### Spectroscopy

For spectroscopic experiments, dark-adapted protein in Tris-buffered solution (pH 7.8) was concentrated to ca. 1 mM for RR and IR difference spectroscopy and to ca. 10 mM for the IR measurements in the nitrile stretching region. For the latter, the sample was additionally semidried to form a 4 μm thick protein film that was sandwiched between two CaF_2_ plates. Prior to the experiments, the sample was fully converted to the Pfr state by illumination with a 670 nm LED. RR measurements were performed using the Bruker Fourier-transform Raman spectrometer RFS 100/S with 1064 nm excitation (Nd:YAG cw laser, line width 1 cm^–1^) equipped with a nitrogen-cooled cryostat from Resultec (Linkam). All spectra of the samples in frozen solution were recorded at ca. 78 K with a laser power of 680 mW at the sample and an accumulation time of typically 1 h. Potential laser-induced damage of the phytochrome samples could be ruled out because comparison of RR spectra before and after a series of measurements did not reveal any changes. For the photoconversion, the protein sample was brought to a required temperature^20^ (e.g., to 293 K to obtain Pr) and illuminated with a 780 nm laser diode for 3–5 min. The illumination time was varied to achieve possibly full conversion. After that, the sample was cooled again to 90 K for measurement. Residual contributions from the nonphotoconverted state were removed by manually weighted spectra subtraction using the OPUS software (Bruker). IR spectroscopic measurements were carried out using a Bruker Tensor 27 FTIR spectrometer in transmission mode. Measurement temperatures were chosen so that the target photoconversion products were stabilized. IR spectra were recorded either in the dark-adapted state or under continuous illumination with a 780 nm LED array. The difference spectra were obtained by 1:1 subtraction of the initial state spectrum from the illuminated state spectrum. Ultraviolet–visible (UV–vis) absorption measurements were performed using a Varian Cary 50 Bio UV–vis spectrophotometer (Agilent) (Figure S2). The protein sample was diluted to an OD at 750 nm of ∼0.25.

### Calculations

The initial structural models for the Agp2 variants F192pCNF and Y165pCNF were generated based on the crystal structure of WT Agp2-PCM in the Pfr state (PDB entry 6G1Y).^27^ The models for the F192oCNF and Y165oCNF variants were directly built from the crystallographic structures. The Lumi-F state was approximated by simply rotating ring D of the BV chromophore (Figure S1) by 180° under the assumption that the photoisomerization occurs on a much faster time scale than the protein relaxation. Missing segments in the amino acid sequence were filled in via 3D homology using SWISS-MODEL^43^ to match the amino acid sequence of Agp2. Single point mutation was done by replacing the respective amino acids at positions 165 and 192 with the Stark reporter group pCNF, respectively. Hydrogens were added to the crystallographic structures according to Karlsberg2+.^44^ The propionic side chain C (propC) of BV was manually protonated following prior spectroscopic studies;^19^ the nitrogen of the pyrrole rings A, B, C, and D was likewise protonated in the Pfr and the Lumi-F state. The protonation states of the amino acids His248 and His278 were modeled as charge-neutral with protons at the ε-position. These starting geometries were solvated in a cubic water box containing TIP3P^45^ water molecules and ionized with sodium and chloride for a neutral system. Energy minimizations were carried out over 40,000 steps using the conjugated gradient algorithm. Subsequent heating to 300 K as well as thermal equilibration was performed with constraints on all heavy atoms, which were gradually released over a time of 90,000 steps. The following 50 ns production run was performed under periodic boundary conditions in an NPT ensemble with a standard pressure of 1.01325 bar, realized with the Langevin piston algorithm,^46^ using the NAMD-V2.10 software.^47^ Electrostatic and van der Waals interactions were cut off at 12 Å, and long-range electrostatic interactions were computed using the particle mesh Ewald (PME) summation method. The protein matrix and the solvent molecules were described using parameters of the CHARMM36 force field.^48^ The BV cofactor was described by parameters taken from a previous work.^49^ The electric field of 1500 frames was computed over the course of the last 30 ns of the 50 ns MD simulation according to Coulomb’s law by projecting the electric field contributions of all point charges surrounding the Stark label onto the nitrile bond vector. The resulting electric fields at the nitrile site were correlated to the number of hydrogen bonds formed with the reporter group. Hydrogen bonds (H-bonds) were identified using the Hbond tool of the VMD program^50^ by assuming a maximal donor–acceptor distance of 3 Å, and the donor–hydrogen–acceptor angle should deviate less than 30° from a linear 180° configuration. The overall distribution of the hydrogen bond–electric field correlation was obtained by applying a kernel density estimation (KDE) performed using Python’s Seaborn library. Basically, the probability density function of the data set was estimated by placing a kernel function, in this case, a Gaussian function, on each data point. The contribution of all kernels at each point was summed and averaged over the number of data points. The smoothness of the resulting density estimate was controlled by the bandwidth that was chosen according to Scott’s rule. Representative frames from each H-bond category were selected according to KDE to ensure a balanced representation of all H-bond configurations without bias. The selected frames served as starting structures for subsequent QM/MM geometry optimization. For these calculations the BV chromophore, the side chains of Cys13, Asp196, and pCNF/oCNF in their respective positions, as well as the pyrrole water, were included in the QM partition and were treated at the B3LYP/6-31g* level of theory. The protein environment, solvent water, and ions were described at a molecular mechanical level using the CHARMM36 force field while only allowing atoms within 20 Å radius around the N22 of BV to move during the energy minimization. Coupling of the QM and MM region was realized with the charge-shifted scheme in combination with the electrostatic embedding approach.^51^ The QM/MM-optimized geometries were further used as input for subsequent frequency calculations of exclusively the QM fragment. These computations were performed at the B3LYP/6-31G* level of theory using GAUSSIAN09^52^ following the same protocol as described previously.^53^ Scaling of force constants, normal-mode analysis, and correction of the QM Hessian matrix were performed using programs developed in our group. The nitrile stretching frequencies were scaled by a factor of 0.953. This factor was derived from the comparison of the calculated and experimental frequencies for various benzonitrile/solvent systems (hexane, DMSO, ethanol, acetonitrile, and THF).^23^ The frequency calculations for these solvent systems were performed at the B3LYP/6-31G* level of theory while treating the solvent implicitly via the polarizable continuum model using the GAUSSIAN09 software. The total electric field of the respective models (F192pCNF, F192oCNF, Y165pCNF, and Y165oCNF) were obtained after re-evaluating the H-bond status and summing up the weighted electric fields according to their relative occurrence during the MD trajectory.

## Results

### Structures of the Parent Pfr States

**Figure 1:**
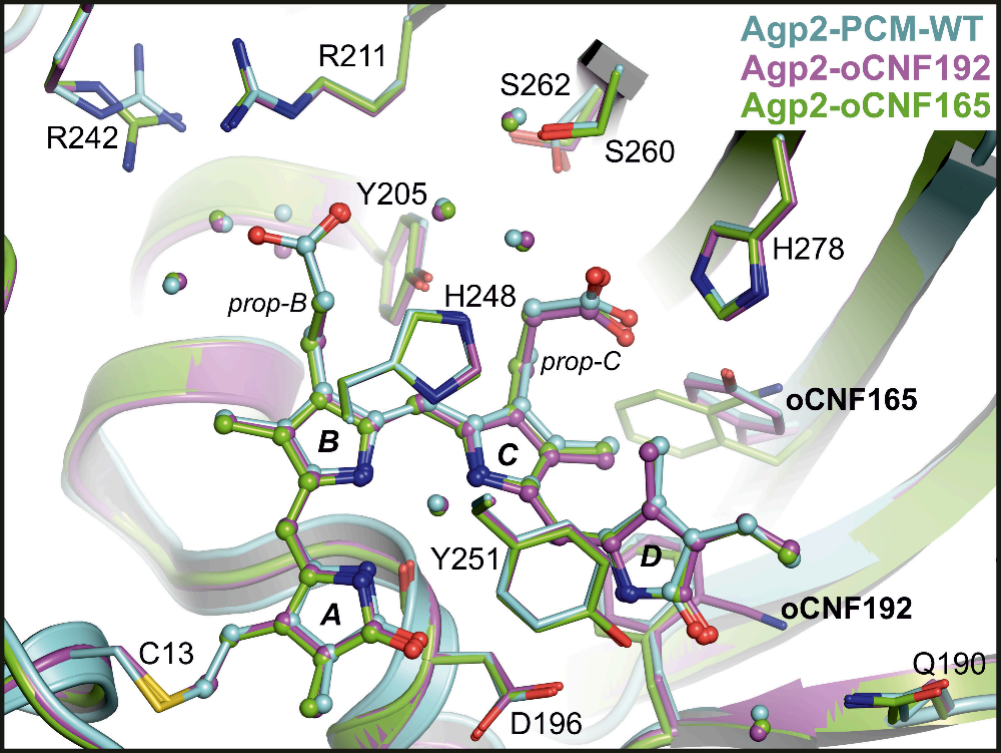
View of the chromophore binding pockets of F192oCNF (magenta, PDB entry 9G8D), Y165oCNF (green, PDB entry 9G8C), and WT Agp2-PCM (cyan, PDB entry 6G1Y) crystal structures of the Pfr states. The protein backbone, chromophore, and selected residues are depicted as cartoon, sticks/balls, and sticks, respectively.

We determined the crystal structures of the Pfr state of Y165oCNF and F192oCNF variants with 1.90 and 2.06 Å resolution, respectively. Superposition with the structure of WT Agp2-PCM by the equivalent Cα atoms yielded a very good alignment with a root-mean-square deviation (RMSD) of only 0.22 Å (Figure 1, Figure S3). Structural details of the *ZZEssa* chromophore geometry and rotamer positions of amino acids within the CBP display a very high similarity between the oCNF variants and the WT protein,^27^ whereby F192oCNF is virtually indistinguishable from the WT. For Y165oCNF, the residues Phe167 and Leu274 are slightly shifted away from the nearby oCNF165 by 0.9 and 0.8 Å, respectively, due to the larger space occupied by oCNF compared to the native Tyr165. Despite this minor difference, the BV chromophore structure remained unaffected by the Y165oCNF substitution, showing nearly perfect alignment with the WT Agp2-PCM.

### Resonance Raman and IR Difference Spectroscopic Characterization

**Figure 2:**
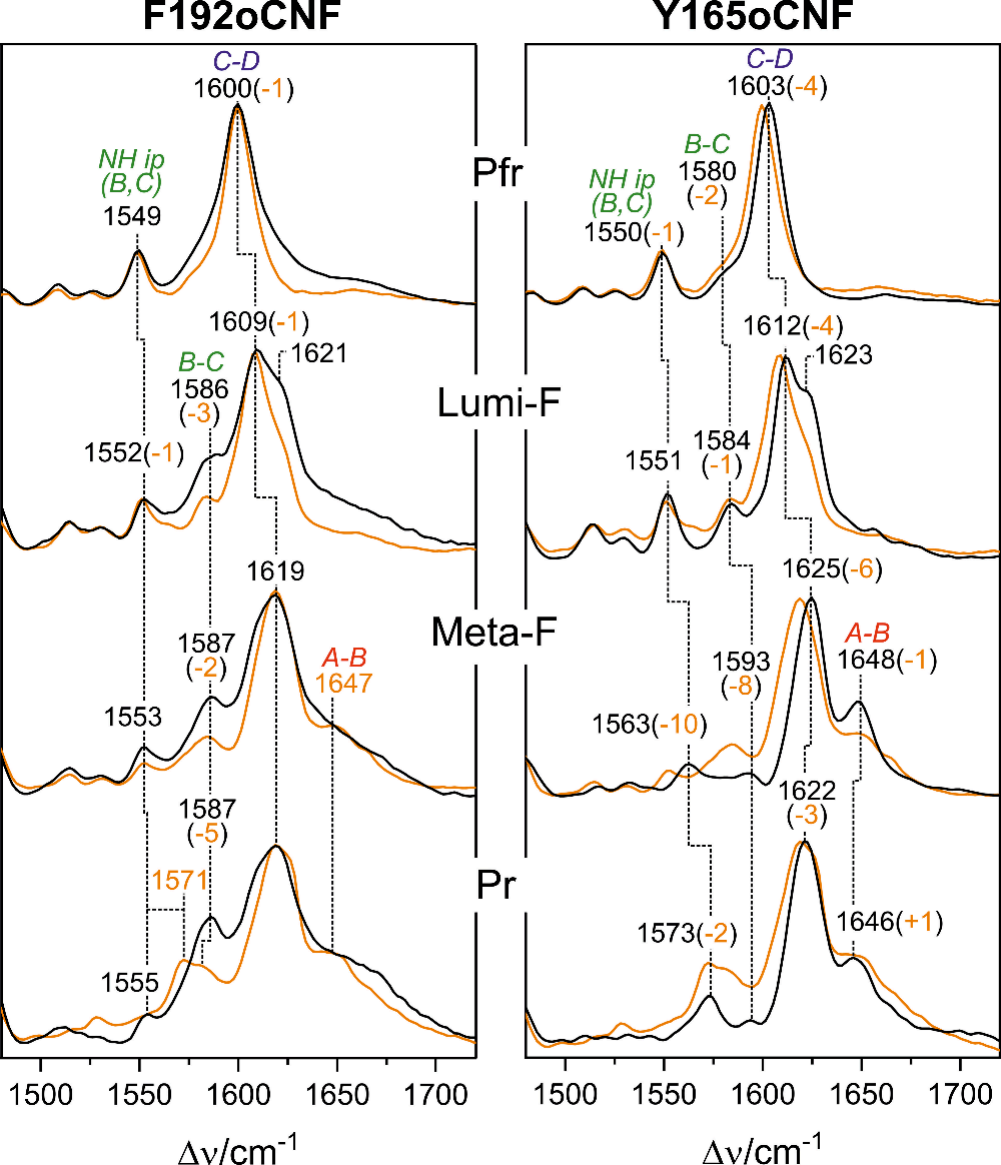
Section of RR spectra of the various states of the Pfr → Pr photoconversion of F192oCNF (left) and F165oCNF (right) (black traces) compared to the WT protein (orange traces, taken from previous work^19,20,23^). Frequency shifts of the WT protein are given in parentheses. The abbreviations A–B, B–C, C–D, and N–H ip (B, C) refer to the stretching modes of the A–B, B–C, and C–D methine bridges and the N–H in-plane bending of rings B and C, respectively. Complete spectra are shown in Figures S4 and S5.

The region between 1500 and 1700 cm^–1^ is dominated by the C=C stretching modes of the methine bridges between pyrrole rings, which are particularly sensitive toward geometrical changes of the tetrapyrrole skeleton (Figure 2; Figures S4 and S5).^54^ In the Pfr state, a nearly perfect agreement between the WT and F192oCNF spectra in this region underpins the preservation of the chromophore structure in both proteins. This conclusion is largely valid also for Y165oCNF, although the stretching of the C–D methine bridge (C–D) displays a 4 cm^–1^ upshift, which suggests a slightly larger dihedral angle and thus stronger torsion of ring D than in the WT. These findings agree very well with the comparison of the crystal structures. Also, the spectra of the Lumi-F state are very similar when comparing the respective spectra of both variants and the WT Agp2-PCM.^19,20,22,23,27,54−56^ A minor exception is again a small upshift of the C–D stretching in Y165oCNF as in Pfr, suggesting that the structural perturbation around ring D persists throughout the photocycle, as also seen by the similar upshifts of this mode in Meta-F and Pr. This, however, does not have any impact on the final photoconversion product Pr, which in the WT protein forms an enol–keto tautomeric equilibrium. As shown previously, the distribution between enol and keto tautomer responds sensitively to substitutions in the CBP.^20,23^ In Y165oCNF, the spectrum is dominated by the keto form, whereas in F192oCNF, the enol tautomer prevails as indicated by the increased intensity at 1250 cm^–1^ (Figure S4) and 1587 cm^–1^ and the absence of the N–H in-plane bending of rings B and C typically observed at ca. 1570 cm^–1^ (Figure 2).^21,54^ In this respect, F192oCNF behaves similarly to F192pCNF and Y165pCNF studied in our previous work,^23^ consistent with the decrease of the Q-band in the UV–vis absorption spectra (Figure S2) and the IR difference spectra discussed below.

**Figure 3:**
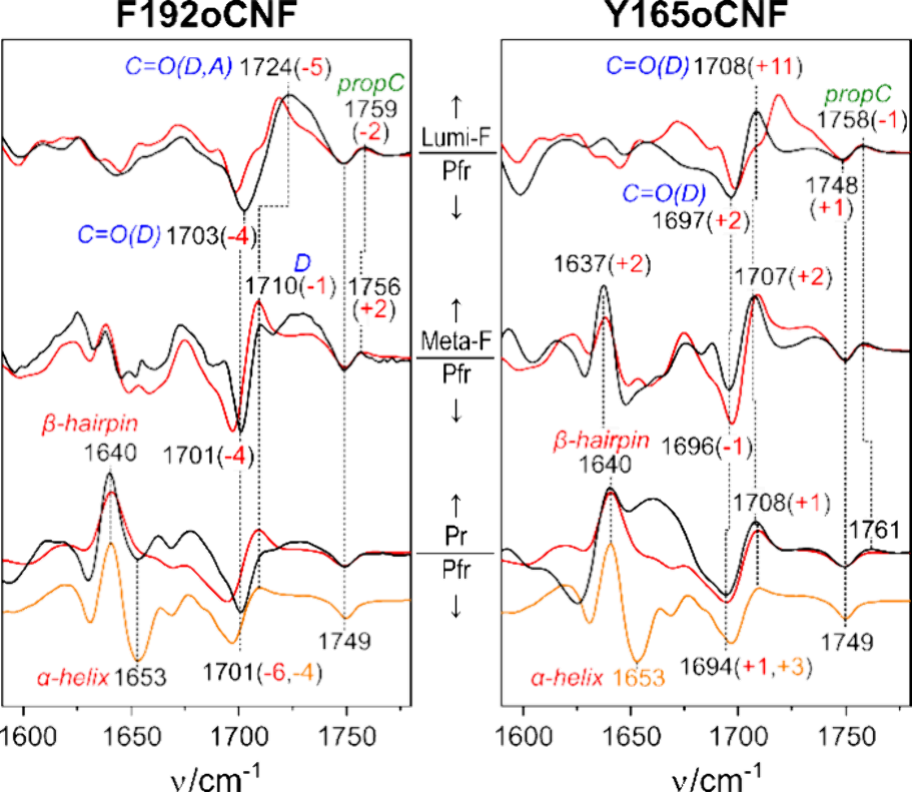
IR difference spectra of the various steps of the Pfr → Pr photoconversion of F192oCNF (left column) and F165oCNF (right column), both displayed by the black traces, compared with those of the WT protein (PCM, red traces). The Pr–Pfr difference spectra (bottom) are further compared to the WT full-length Agp2 difference spectrum (orange trace) that shows a distinctly slower thermal Pr → Pfr back-conversion than WT Agp2 PCM and hence more pronounced signals of the tongue restructuring (α-helix → β-sheet).^20,23^

The IR difference spectra of the two variants are also closely related to those of the WT protein (Figure 3). Whereas the Lumi-F-minus-Pfr and Meta-F-minus-Pfr difference spectra show no signals in the amide I band region, the Pr-minus-Pfr difference spectra display a strong positive signal at ca. 1640 cm^–1^ and a smaller negative peak at ca. 1655 cm^–1^, reflecting the transition from an α-helix (negative peak) to a β-sheet structure (positive peak).^19,20,23^ This signal pair indicates the restructuring of the tongue, which is linked to the deprotonation of propC as demonstrated by the negative signal at ca. 1750 cm^–1^ and the lack of a positive counterpart. In Lumi-F and Meta-F, propC remains protonated, as reflected by the positive and negative signal pair at ca. 1750/1760 cm^–1^. Again, Y165oCNF behaves slightly differently because the signal at 1760 cm^–1^ does not completely disappear in Pr, pointing to residual faction of protonated propC. This is accompanied by an additional broad positive signal at ca. 1660 cm^–1^ that obscures the negative signal at 1653 cm^–1^ originating from the α-helical tongue segment. A notable frequency shift is observed for the ring D carbonyl group in Y165oCNF, particularly in the Lumi-F state, thereby mirroring the small spectral changes in ring D noted in the RR spectra. In the Pr-minus-Pfr difference spectrum of F192oCNF, the lack of a positive band in this region confirms the shift of the tautomeric equilibrium toward the enol form. A small enol content might also exist in the Meta-F state in view of the lower intensity of the band at 1710 cm^–1^.

### Experimental Analysis of the Nitrile Stretching Mode

**Figure 4:**
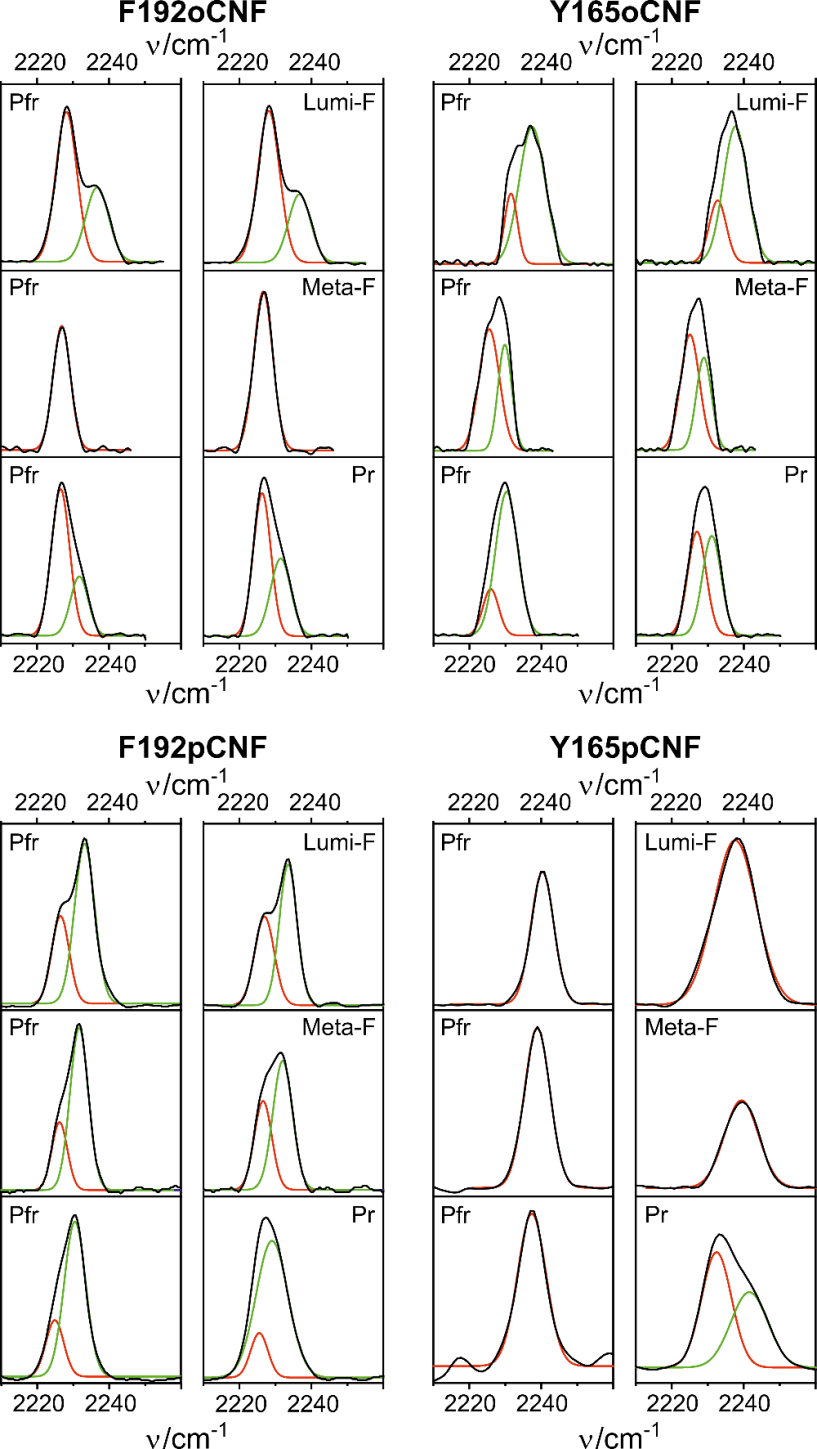
IR spectra of the nitrile stretching mode of the four cyanophenylalanine-substituted Agp2 variants. Experimental spectra are depicted in black, and the fitted Gaussian bands are in green and red. Data for Y165pCNF and F192pCNF were taken from a previous work.^23^ Vibrational frequencies are listed in Table S2.

IR spectra in the region of the nitrile stretching modes were measured at various temperatures to trap the intermediates Lumi-F and Meta-F and the final photoproduct Pr. Prior to irradiation at each trapping temperature, the spectrum of Pfr was measured. After baseline correction, the spectra were analyzed via band fitting with one or two Gaussian functions to evaluate the integral intensities and frequencies of the individual components. The analyzed spectra of F192oCNF and Y165oCNF are shown in Figure 4 together with those of F192pCNF and Y165pCNF reported previously.^23^

The nitrile stretching frequencies are temperature-dependent, and the variations are particularly strong for nitrile groups involved in H-bonding interactions.^57^ To ensure comparability between the spectra of the various states, we first assume that the temperature dependencies, as determined for the two nitrile band components of Pfr, are the same for the photoproducts (PP). Hence, the frequencies were normalized to Pfr measured at room temperature (RT, 300 K) by maintaining the frequency difference (Pfr minus PP) of the conjugate band components (Table S2). Second, also the integral intensities were normalized to Pfr at 300 K (RT) according to
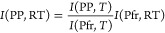
1where *I*(PP, *T*) and *I*(Pfr, *T*) are the intensities of the photoproduct and Pfr at the trapping temperature (Table S3).

### Analysis of the Total Electric Field

The starting point for the analysis of the electric field changes during the photocycle was the Pfr state because reliable structural data were available and could be used for MD and QM/MM calculations. The crystal structures of the F192oCNF and Y165oCNF Pfr states were obtained in the present work and described above. For the corresponding pCNF variants, structural models based on the crystal structure of the WT Pfr were obtained by *in silico* amino acid substitution.^23^ The geometry-optimized structures of all four models reveal far-reaching similarities with the WT Pfr (*vide supra*; Figure S6). MD simulations were employed, and after equilibration, 50 snapshots were selected for which QM/MM calculations were carried out. The computed Raman spectra show a very good agreement with the experimental spectra (Figure S7), underscoring the high quality of the structural models and the theoretical approach. Additional parameters evaluated for the Pfr state of each variant during the QM/MM calculations included the transition dipole moment (TDM) of the nitrile stretching mode  and the electric field projected onto the nitrile group *E*_F,tot,Pfr_. Because both quantities were obtained by averaging over 50 QM/MM snapshots, they refer to the total population of the Pfr state.

The electric field of the total Pfr population, *E*_F,tot,Pfr_, is related to the TDM  via
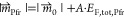
2where  is the TDM of the nitrile stretching in the absence of an electric field and *A* is a constant.^16,58^ These two parameters were then determined by a linear fit to the TDM/electric-field plot of the data from the individual snapshots (Figure S8) according to eq 2. Whereas the intercept  was characteristic of the type of Stark effect label (i.e., oCNF or pCNF), the slope *A* was the same for all variants, i.e.,  (Table 1).

**Table 1:** Normalized Intensities, Frequencies, and Transition Dipole Moments^a^ Estimated for Spectral Analysis

variant			Pfr	Lumi-F	Meta-F	Pr
Y165oCNF	band envelope	*I*(RT)_tot_	7.64	7.484	7.028	7.63
		,	7.767	7.687	7.449	7.762
	peak P1	ν	2225.9	2227.1	2225.4	2226.9
		*I*(RT)_1_ · *x*_1_	1.11	1.832	4.392	3.72
			2.960	3.802	5.889	5.428
	peak P2	ν	2230.4	2230.7	2229.4	2231.0
		*I*(RT)_2_ · *x*_2_	6.5	5.652	2.635	3.91
			7.181	6.680	4.561	5.556
F192oCNF	band envelope	*I*(RT)_tot_	12.86	12.70	9.130	14.04
		,	6.196	6.157	5.220	6.474
	peak P1	ν	2226.6	2226.6	2226.9	2226.3
		*I*(RT)_1_ · *x*_1_	9.22	8.746	9.130	8.61
			5.246	5.110	5.220	5.070
	peak P2	ν	2231.8	2231.7		2231.5
		*I*(RT)_2_ · *x*_2_	3.64	3.955		5.43
			3.296	3.436		4.026
Y165pCNF	band envelope	*I*(RT)_tot_	2.642	6.172	1.934	3.780
		,	9.564	14.618	8.183	11.44
	peak P1	ν				
		*I*(RT)_1_ · *x*_1_				
				
	peak P2	ν	2237.4	2234.7	2237.9	2232.4
		*I*(RT)_2_ · *x*_2_	2.642	6.172	1.934	2.094
			9.564	14.168	8.183	8.515
	peak P3	ν				2241.6
		*I*(RT)_3_ · *x*_3_				1.686
						7.640
F192pCNF	band envelope	*I*(RT)_tot_	1.516	1.346	1.532	1.796
		,	8.829	8,319	8.875	9.610
	peak P1	ν	2224.9	2225.3	2225.1	2225.5
		*I*(RT)_1_ · *x*_1_	0.352	0.549	0.582	0.268
			4.254	5.313	5.470	3.712
	peak P2	ν	2230.5	2230.8	2230.8	2229.0
		*I*(RT)_2_ · *x*_2_	1.164	0.797	0.950	1.528
			7.736	6.401	6.989	8.864

a Intensities *I*(RT) are given in arbitrary units, frequencies ν in cm^–1^, and transition dipole moments  in (km · mol)^1/2^. Note that for the individual peaks, the listed values of the intensities and transition dipole moments include the factor *x*_*i*_ and , respectively (see eqs 7 and 8). The transition dipole moment at zero field, , is 4.44 and 7.04 (km · mol)^1/2^ for the oCNF and pCNF variants, respectively. The transition dipole moments of the band envelopes of Pfr () were directly calculated on the basis of the QM/MM structural models (see eq 2). This quantity was used to evaluate the () values of the photoproducts based on the intensities (eq 3).

Because the TDM is proportional to the square root of the integral intensity,  refers to the intensity of the total population of the Pfr state, i.e., to the sum of all band component intensities. Consequently,  serves as an internal standard for calculating the TDMs of the photoproducts (Lumi-F, Meta-F, and Pr) . After normalization of the intensities to Pfr at 300 K (RT), one obtains
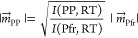
3

To evaluate the total electric field *E*_F,tot,PP_ for the photoproducts, the respective eq 4
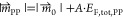
4is divided by eq 2 to obtain
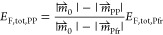
5

The results are given in Table 2. Note that the QM/MM-calculated quantities were restricted to *E*_F,tot,Pfr_ and  and thus, via eq 2,  and *A*. The evaluation of all other quantities in this work required the use of experimental parameters (frequencies, intensities).

**Table 2:** Electric Fields of the Individual Populations of the Four Variants

	*E*_F,tot_^a^^,^^c^	*x*_1_	*x*_2_	*x*_3_	*E*_F,1_^a^^,^^d^	*E*_F,2_^a^^,^^e^	*E*_F,3_^a^^,^^e^	b	b	b	*E*_F,sum_^a^^,^^f^	RMSD^g^
Y165oCNF												
Pfr	–53.41	0.25	0.75		–32.09	–81.68		5.948	8.279		–69.40	29.93
Lumi-F	–52.13	0.44	0.56		–27.61	–95.34		5.738	8.921		–65.58	25.82
Meta-F	–48.30	0.95	0.05		–33.96	–347.8		6.036	20.785		–49.07	1.58
Pr	–53.33	0.88	0.12		–28.36	–252.7		5.773	16.318		–54.37	1.95
F192oCNF												
Pfr	–45.40	0.81	0.19		–29.48	–66.8		5.826	7.580		–36.54	19.52
Lumi-F	–44.39	0.77	0.23		–29.48	–57.78		5.826	7.156		–36.01	18.89
Meta-F	–20.17	1.0			–28.36			5.220			–28.36	40.60
Pr	–52.59	0.74	0.26		–30.60	–74.81		5.878	7.956		–41.92	20.29
Y165pCNF												
Pfr	–73.84		1.00			–53.70			9.564		–53.70	27.27
Lumi-F	–221.70		1.00			–161.23			14.618		–161.23	27.27
Meta-F	–33.44		1.00			–24.32			8.183		–24.32	27.27
Pr^h^	–128.72		0.36	0.64		–152.16	–53.40		14.192	9.550	–88.96	30.89
F192pCNF												
Pfr	–28.76	0.24	0.76		–35.82	–38.74		8.724	8.861		–38.05	32.30
Lumi-F	–20.56	0.38	0.62		–34.33	–22.75		8.654	8.109		–27.12	31.89
Meta-F	–29. 50	0.40	0.60		–35.07	–41.61		8.688	8.996		–39.02	32.26
Pr	–41.32	0.19	0.81		–33.58	–59.19		8.618	9.822		–54.44	31.76

a In MV/cm.

b In

c Calculated for the Pfr states by QMMM; the values for the other states were obtained via eq 5.

d Evaluated from the frequency shift (eq 11); considered to be of purely noncovalent character.

e Evaluated from the intensities as described in the text; originating from H-bonding and noncovalent effects.

f Sum of *x*_1_*E*_F,1_, *x*_2_*E*_F,2_, and *x*_3_*E*_F,3_.

g In percent. The total RMSD was 26.8%.

h In an alternative solution, the electric fields of the P2 and P3 and the mole fractions were nearly reversed. The error was exactly the same.

Additionally, we determined  and *E*_F,tot,Lumi_ directly from structural models. A Lumi-F-like state was generated from the respective structures of the Pfr states by *in silico* rotation of the C–D methine bridge and subsequent MD and QM/MM optimization. The values of the directly calculated TDMs and electric fields deviate substantially in both directions (±46%) from the values derived from the experimentally determined intensities via eqs 1–5 (Table S4). Presumably, the *in silico* isomerization of the chromophore and subsequent structural optimization of the protein did not lead to sufficiently accurate models for reliable electric field and TDM calculations. This again demonstrates the importance of high-quality structural models for a reliable QM/MM calculation. Interestingly, the Raman spectra calculations do not seem to respond similarly sensitively to uncertainties in the structures because calculated spectra of Lumi-F provide descriptions of the experimental spectra that are almost as good as for Pfr (Figure S7).

### Analysis of the Electric Fields of Individual Substates

In most cases, there are two closely spaced band components of the nitrile stretching mode that can readily be identified by visual inspection and quantitatively analyzed by a band fitting. The sum of the electric fields of the individual substates *E*_F,*i*_, weighted by the respective mole fractions *x*_*i*_, yields
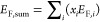
6

These values should match the electric fields derived from the total population of Pfr models, *E*_F,tot,Pfr_, as well as those obtained for each photoproduct using eq 5, *E*_F,tot,PP_ (Table 2).

The normalized intensities of conjugate band components *I*(RT)_*i*_ (*vide supra*, Table 1) are related to the total intensity *I*(RT)_tot_ via the mole fractions *x*_*i*_ by
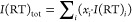
7

Hence, the determination of the TDMs for the individual band components *i* according to eq 3 must consider the relative populations *x*_*i*_ that give rise to component intensities *I*(RT)_*i*_. Thus, applying eq 3 to the individual band components does not yield the respective individual TDMs but the products
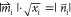
8which are listed in Table 1. Accordingly, determination of the electric fields related to the band components *E*_F*,i*_ (substates) was carried out via a modification of eq 2, i.e.,
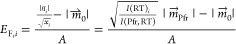
9

Note that estimation of *E*_F,*i*_ requires empirical input (*I*(RT)_*i*_, *I*(Pfr, RT)) as well as parameters derived from computations ,  and *A*).

Furthermore, the underlying substates of the nitrile groups typically differ with respect to the relative contributions of noncovalent and H-bonding electric field (*vide infra*). These two effects can be disentangled on the basis of the intensities and frequencies.

The frequencies of the individual components ν_*i*_ are related to the electric field but additionally depend on H-bonding interactions.^6,14−16^ These two effects show dependencies in opposite directions. Nitrile groups in an environment free of H-bonding interactions are described by the linear VSE
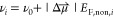
10or with Δν_non_ = ν_*i*_ – ν_0_
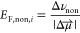
11where  is the Stark tuning rate, which for QM/MM-calculated fields of benzonitrile in various solvents was shown to be ca.  = 0.268 (cm/MV)·cm^–1^.^23^ For the zero-field frequency ν_0_, one may take the value measured for benzonitrile in the gas-phase, i.e., ν_0_ = 2234.5 cm^–1^.^59^ The effect of H-bond interactions will be discussed latter.

### Evaluation of the Electric Fields in the Individual Substates

In all states of the F192oCNF, Y165oCNF, and F192pCNF variants, one band component (denoted as P1 in all tables) was detected with a frequency below 2227 cm^–1^, which was attributed to a nitrile in a hydrophobic environment (*vide infra*). For this component, the electric field was determined via eq 11 (Table 2) based on the experimental nitrile stretching frequency ν_*i*_. All other band components (denoted as P2 and P3) refer to nitrile groups involved in H-bonding interactions, and the respective electric fields were evaluated via eq 9 using the corresponding normalized intensity *I*(RT)_*i*_. Y165pCNF is a special case because for Pfr, Lumi-F, and Meta-F, only one component was found, but there were two for Pr. As judged from the frequencies, all components are involved in H-bonding interactions. For the two components P2 and P3 of Pr, there was no unique solution on the basis of eq 9 (Table 2).

The accuracy of the procedure was checked by calculating the sum of the electric fields *E*_F,sum_ of each state according to eq 6 and relating these quantities to the calculated values derived from the Pfr models (*E*_F,tot_). The overall RMSD was determined to be 26.8%. Large underestimations of the electric fields were noted for the strongly H-bonded components and in those cases where the broad band envelope did not allow for an unambiguous identification of two components (Meta-F of F192oCNF) (Table 2).

### Electric Fields Due to Noncovalent and Hydrogen-Bond Interactions

In the following, we consider only peaks P2 and P3, which represent nitrile groups involved in H-bonding interactions. In this case, the electric field *E*_F,*i*_ sensed by the stretching mode may be divided into the effect of the H-bonding *E*_F,HB,*i*_ in the “primary coordination sphere” of the nitrile and the noncovalent contribution *E*_F,non,*i*_ provided by the dipoles and charges of the environment in the “second coordination sphere”:
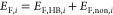
12

This also implies that the observed frequency shift is the sum of the noncovalent and H-bonding induced shifts
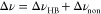
13or with eq 11
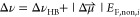
14

A similar substitution can be made for Δν_HB_ to give
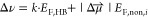
15although the factor *k* is not known a priori. However, it may be approximated on the basis of an empirical relationship derived by Deb et al. for benzonitrile in aqueous solutions of organic solvents,^60^ i.e.,
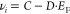
16where *C* and *D* are positive constants that depend on the kind of method for electric field calculations. The reported value for *D* using classical MD-calculated fields was −0.317 (MV/cm)^−1^ cm^–1^,^60^ which was scaled to our QM/MM-calculated fields on the basis of the calculated fields for benzonitrile in pure water. Thus, we obtained −0.2 (MV/cm)^−1^ cm^–1^, which we took as an approximate value for the factor *k* in eq 15. Combining eqs 12 and 15 then yields
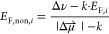
17

The results are listed in Table 3. Within the underlying assumptions of eqs 12 and 16, the overall error of the separation into noncovalent and H-bond electric fields is assumed to be similar to the error of the determining the overall fields for each substate (i.e., ca. 25%, Table 2). Exceptions are, most likely, the values for peak 2 of Meta-F and Pr in Y165oCNF where the very small mole fraction seem to be associated with unrealistically high fields.

**Table 3:** Noncovalent and H-Bonding Electric Fields

	*E*_F,sum_^a^^,^^b^	P1		P2				P3				*E*_F,tot,non_^a^^,^^g^	E_F,tot,HB_^a^^,^^h^
		*x*_1_	*E*_F,1_^a^^,^^c^	*x*_2_	*E*_F,2_^a^^,^^d^	*E*_F,2,non_^a^^,^^e^	*E*_F,2,HB_^a^^,^^f^	*x*_3_	*E*_F,3_^a^^,^^d^	*E*_F,3,non_^a^^,^^e^	*E*_F,3,HB_^a^^,^^f^		
Y165oCNF													
Pfr	–69.40	0.25	–32.09	0.75	–81.68	–43.67	–38.01					–40.80	–28.60
Lumi-F	–65.58	0.44	–27.61	0.56	–95.34	–48.86	–46.48					–39.53	–26.06
Meta-F	–49.07	0.95	–33.96	0.05	–347.8	–159.52	–188.25					–40.00	–9.06
Pr	–54.37	0.88	–28.36	0.12	–252.7	–115.48	–137.25					–38.46	–15.91
F192oCNF													
Pfr	–36.54	0.81	–29.48	0.19	–66.8	–34.32	–32.49					–30.40	–6.14
Lumi-F	–36.01	0.77	–29.48	0.23	–57.78	–30.67	–27.10					–29.76	–6.25
Meta-F	–28.36	1.0	–28.36									–28.36	0.0
Pr	–41.92	0.74	–30.60	0.26	–74.81	–38.38	–36.43					–32.59	–9,33
Y165pCNF													
Pfr	–53.70			1.00	–53.70	–16.75	–36.95					–16.75	–36.95
Lumi-F	–161.23			1.00	–161.23	–68.48	–92.76					–68.48	–92.76
Meta-F	–24.32			1.00	–24.32	–3.13	–21.19					–3.13	–21.19
Pr^i^	–88.96			0.36	–152.16	–69.51	–82.65	0.64	–53.40	–7.65	–45.75	–29.92	–59.04
F192pCNF													
Pfr	–38.05	0.24	–35.82	0.76	–38.74	–25.10	–13.64					–27.65	–10.40
Lumi-F	–27.12	0.38	–34.33	0.62	–22.75	–17.63	–5.12					–23.92	–3.19
Meta-F	–39.02	0.40	–35.07	0.60	–41.61	–25.69	–15.92					–29.41	–9.61
Pr	–54.44	0.19	–33.58	0.81	–59.19	–37.05	–22.14					–36.40	–18.03

a In MV/cm.

b Sum of the electric fields evaluated for all components.

c Evaluated from the frequency shift (eq 11); considered to be of purely noncovalent character.

d Including H-bonding and noncovalent effects.

e Noncovalent fields.

f H-bonding fields.

g Sum of *x*_1_*E*_F,1_, *x*_2_*E*_F,2,non_, and *x*_3_*E*_F,3,non_.

h Sum of *x*_2_*E*_F,2,HB_ and *x*_3_*E*_F,3,HB_.

i For the alternative solution (see Table 2), the electric fields of the population differ by ca. ±10% and the mole fraction differs by ca. ±0.1.

## Discussion

### Environment of the Nitrile Labels

The positions for introducing the CNF labels were chosen such that they were located in the proximity of the BV chromophore. The substitutions should be conservative, which made Tyr and Phe the most suitable candidates, in particular, Tyr165 and Phe192. Specifically, the substitution of these residues by nitrile-substituted phenylalanines should not affect the key structural properties of Agp2 and their changes during the Pfr → Pr photoconversion. This condition is, in fact, fulfilled, as RR spectroscopy demonstrated largely unchanged chromophore structures, and IR difference spectroscopy revealed protein structural changes similar to those in the WT protein. This is true for the ortho-CNF and the para-CNF variants, studied in this and our previous work, respectively.^23^ Specifically, the functionally crucial structural changes are preserved: chromophore photoisomerization at the C–D methine bridge in the primary reaction step, deprotonation of propC, and the concomitant secondary structure transition α-helix to β-sheet during the decay of Meta-F to Pr and the formation of the keto–enol tautomeric equilibrium of the chromophore in Pr. Minor differences that do not affect the functional integrity include a slightly higher torsion of ring D and an increased keto content in the Pr state of Y165oCNF and the reaction kinetics. The latter includes the dark reversion, which was generally slower than in the WT protein (Figure S2). Differences in the activation barriers of the decay processes of Meta-F may account for slightly different distributions among its cryotrapped substates, as reflected by small deviations in the C–D stretching frequencies.^19,21,27,54^ In summary, we conclude that nitrile probes at positions 165 and 192 do not impair the crucial molecular steps of the phototransformation although structural details may be different, specifically in Y192oCNF.

However, the involvement of residues Tyr165 and Phe192 in the photocycle of Agp2 is different, and thus, the respective nitrile probes might sense different electric field effects. Tyr165 is essential for the proton transfer from propC in the final step of the photoconversion from Meta-F to Pr.^20^ Phe192 is a part of the steric interaction cascade in the CBP that links the rotation of ring D to the restructuring of the tongue (WT Agp2-PCM, PDB entry 6G1Y).^27^ In particular, H-bonding interactions are different for these residues. Phe192 is located in a hydrophobic part of the CBP. Accordingly, no strong H-bonds are expected for either pCNF or oCNF at position 192, which is confirmed by the crystal structure (F192oCNF) and the structural model (F192pCNF) for the Pfr state with the exception of a H-bond in the oCNF192 backbone to Gly173. This lack of significant H-bonding interactions is most likely also true for the other states of these variants because the nitrile stretching frequencies are relatively low in each case. On the contrary, Tyr165 in WT Agp2 is H-bonded to propC and His278 in Pfr.^27^ It is reasonable to assume that it remains H-bonded throughout the photocycle, although the H-bonding partners may change to C=O(D) and/or His278 in Lumi-F (in analogy to the related bacteriophytochrome from *Pseudomonas aeruginosa*),^61^ Arg202 via water molecules in Meta-F (Agp2-PCM variant PAiRFP2, PDB entry 6G20),^27^ and Pr (in analogy to Agp1-PCMser13, PDB entry 5HSQ).^62^ Similar interactions may be assumed for Y165pCNF, thanks to the same orientation of the nitrile and hydroxyl substituent in pCNF and Tyr, as confirmed by the structural model for Pfr and the high frequencies of the nitrile stretching in all states. Interactions of the nitrile group in Y165oCNF seem to be more variable because the *ortho* position of the CN label allows for different, possibly coexisting rotamers. Our crystal structure of Y165oCNF in the Pfr state shows that H-bonding differs from that of the WT Agp2-PCM, as the nitrile group is pointing away from the potential H-bonding donors propC and H278 and no H-bonds are found except for a hydrogen bridge of the Y165oCNF backbone (Figure 1). Again, this finding is qualitatively in line with the nitrile stretching frequencies that are in each substate of Y165oCNF distinctly lower than in the Y165pCNF variant. Hence, the four variants allow for a comparison of the electric field at two positions and at each position in two directions within the CBP.

### Electric Field Changes during the Photoconversion

Starting points for the electric field analysis were the structures of the Pfr state, which allowed the evaluation of the total electric field *E*_F,tot_ sensed by the nitrile group (Table 2). Upon comparison of the integral normalized intensities, this quantity could also be determined for the other states of the photoconversion. The overall error of *E*_F,tot_ is estimated to be below 20%. This estimate is derived from the RMSDs of the TDM (12.8%) and *E*_F,tot_ (15.2%) calculations for Pfr and the distinctly smaller error of the intensity determination. Interestingly, there is no uniform tendency of the electric field changes during the photoconversion, and even for the same substitution site but different orientations (*ortho* vs *para*), the nitrile senses substantially different electric field magnitudes. In Y165oCNF, *E*_F,tot_ remains largely constant within the accuracy of the field calculations, whereas in Y165pCNF, we note two extraordinarily high values in Lumi-F and Pr. A strong field increase upon Pr formation is also found for F192pCNF and F192oCNF, which is, however, accompanied by minima in Lumi-F and Meta-F, respectively.

More specific insights into the local electric field distribution can be obtained upon consideration of the individual peak components. Interestingly, the F192oCNF, F192pCNF, and Y165oCNF variants show two clearly identifiable band components in each state, except for Meta-F of F192oCNF that displays a symmetric band shape of the nitrile stretching mode. In all other states, there is one band component with a frequency ≤2227.1 cm^–1^, whereas the second one has a frequency ≥2229.0 cm^–1^. The low- and high-frequency components were ascribed to the nitrile group in the absence and presence of H-bonding interactions, which provided a consistent analysis. This classification is based on the IR measurements of benzonitrile in various water-miscible organic solvents with different water contents covering a frequency range from ca. 2228 (0% water) to ca. 2235.5 cm^–1^ (100% water).^60^ Accordingly, the single band component of Meta-F of F192oCNF (2226.9 cm^–1^) is attributed to a H-bond free species. F192pCNF was a special case. In Pfr, the theoretical simulations did not reveal any H-bonding contact to the nitrile group, whereas the spectrum displayed two nitrile stretching band components with frequencies characteristic of a hydrophobic and a H-bonding environment. We have adopted this scenario for all states of F192pCNF because the alternative assumptions with both band components reflecting H-bonded nitrile groups or both nitrile populations free of any H-bonding interactions yielded partly meaningless results.

In general, the interpretation of the two-component scheme is not unambiguous. First, it is a simplification because there are most likely much more conformational and H-bonded states of the nitrile group that are just sorted out into two main ensembles. A good example is the Meta-F state of F192oCNF with a symmetric band shape that points to a Gaussian-like distribution of substates. In this particular case, however, also the reference spectrum of Pfr at 240 K shows a single band of the nitrile stretching, dissimilar to Pfr at 140 and 300 K (Figure 4). This finding suggests a structural transition of the frozen protein below and above 240 K that is independent of the chromophore state and specific to this variant. Second, the simplified two-component scheme may reflect fluctuating interactions with H-bond donors (i.e., contact on or off) or—as previously suggested for the band doublet in F192pCNF—a conformational equilibrium of the protein involving the CNF residues or a combination of both.^23^

### Hydrogen Bonding and Noncovalent Electric Fields

To understand structural changes and reaction mechanisms in terms of electric field control, it is important to separate noncovalent field contributions from those of H-bonding interactions. Weaver et al. made a first attempt assuming that the observed frequency shift is essentially the sum of the individual frequencies resulting from the two effects.^16^ The authors used the linear relationship in eq 10 as a reference that relates an electric field to a vibrational shift. For a H-bonded nitrile experiencing the same field strength, the observed shift is smaller, and Weaver et al. took the difference to the reference value as the frequency shift due to noncovalent electric field.^16^ This approach implies that noncovalent and hydrogen-bonding contributions are proportional, which in our case led to unrealistically large noncovalent fields when the total frequency shift was small or positive.

Instead of the different frequency shifts Δν_*i*_, we now considered the different field dependencies of the nitrile stretching frequencies  for noncovalent interactions (eq 10) and H-bonded nitriles (eq 16).^60^ However, our approach also includes a simplifying approximation. It tacitly assumes that eq 16, derived from benzonitrile in organic solvents with different water contents,^60^ reflects exclusively hydrogen-bonding effects whereas noncovalent contributions are neglected. An additional underlying assumption, presumably with only a minor impact on the overall error, implies that the distribution of σ- and π-hydrogen bonds, which have different consequences for the nitrile stretching frequency,^63^ is the same for the nitrile group in solution and in the protein.

### Hydrogen Bonding Effects

**Figure 5:**
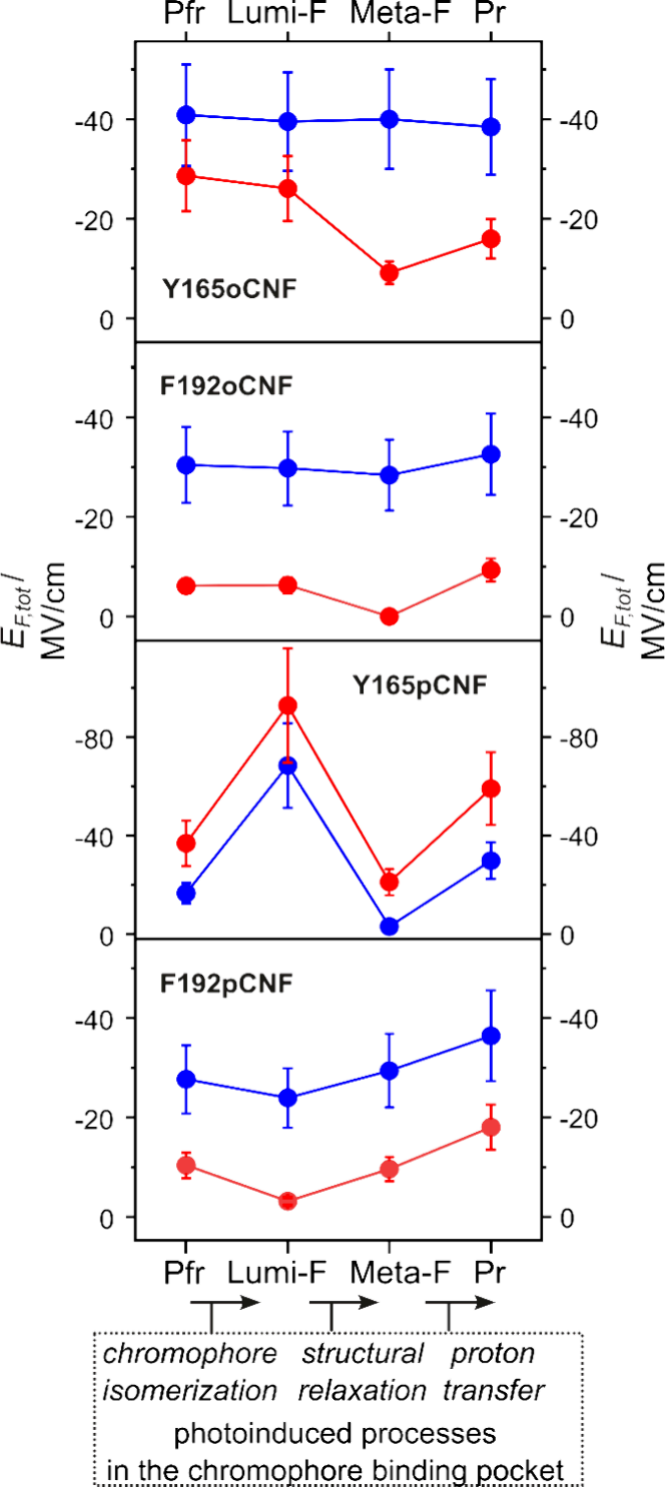
Electric field changes sensed by the nitrile label during the Pfr → Pr photoconversion of F192pCNF, F192oCNF, Y165pCNF, and Y165oCNF. The blue and red traces refer to the noncovalent and H-bonding electric fields, respectively (Table 3).

The strength of H-bonds is usually expressed by the enthalpy of bond formation. In the case of H-bonds to nitrile groups, this quantity may be related to the electric field acting on the nitrile bond, thereby representing an alternative measure of the H-bond strength. For the sake of simplicity, we may therefore classify the H-bonds of the protein-bound phenyl substituents on the basis of the *E*_F,tot,HB_ values as very weak (0–15 MV/cm), weak (15–30 MV/cm), medium (30–45 MV/cm), strong (45–60 MV/cm), and very strong (>60 MV/cm). Both F192pCNF and F192oCNF reveal only very weak H-bonding interactions because this residue is located in an essentially nonpolar pocket as shown by Pfr structures (Figures 1 and 5). This is most likely true also for Lumi-F and Meta-F, and only in Pr is there a notable increase in the H-bond strength. Possibly, the structural changes of the protein upon the Meta-F → Pr transition cause a slight opening of the pocket, allowing water molecules to intrude and interact with the nitrile group. Y165pCNF is the opposite example because H-bonding interactions are much stronger in all states. Again, this finding is not surprising because, in this case, the nitrile just substitutes the hydroxyl group of Tyr165 that in turn is part of the H-bond network in the CBP. In this sense, the data for Y165pCNF may mimic very well the natural changes of the H-bond interactions of Tyr165 during the Pfr → Pr photoconversion. In Pfr, the protonated propC and Tyr165 form a H-bond that in Meta-F is disrupted due to a repositioning of Tyr165 toward Phe192 and the hydrophobic part of ring D.^20,21,27^ Instead, Tyr165 gets involved in a water-mediated H-bond to Arg202, which may account for a lower H-bond strength in Meta-F. In Y165pCNF, this transition from Pfr (medium) to Meta-F (weak) proceeds via a transiently strong increase of the H-bonding interactions in Lumi-F (very strong), which cannot be plausibly explained on the basis of the present calculated Lumi-F model. The final increase of the H-bond strength in Pr (strong) may be possibly due to an entrance of water molecules into CBP. Except for a minimum in Meta-F, this behavior is not mirrored by Y165oCNF because the different orientation of the nitrile group largely impairs the natural H-bond contacts in Pfr (Figure 1) and most likely also in the other states.

In this context, it is interesting to compare the present results with those reported by Kurttila et al., who studied the prototypical phytochrome *Dr*BphP, carrying azido-labels, by time-resolved IR spectroscopy.^64^ The azido group introduced, *inter alia*, at the positions analogous to Tyr165 and Phe192 of Agp2 (Tyr176 and Phe203, respectively) by *para*-azido-phenylalanine (pAzF) gives rise to a N≡N stretching mode around 2100 cm^–1^ that, however, does not display a clear and quantitative relationship with the H-bond strength or the local electric field. Instead, it is considered a qualitative marker for polarity changes, thereby indirectly reflecting the proximity of H-bond donors. In the case of Y176pAzF, changes were only observed at the later stages of the photoconversion, suggesting an increase of the H-bond strength in the final state Pfr. Although the residue is located in a hydrophobic pocket, the azide frequency points to a H-bonded environment. This was attributed to a H-bond with a water molecule that in Pfr is substituted by a stronger H-bond partner. Inserting the label at position 203 revealed the surprising result that the proper formation of Meta-R was hindered, preventing information about possible polarity changes at this position in the native protein.

### Noncovalent Effects

In both Agp2 variants carrying the nitrile group at the *ortho* position, the noncovalent electric fields remain largely constant during the photoconversion (Figure 5). Presumably, the orientation of nitrile groups is unfavorable to sense electric field changes during the photoconversion because, in the corresponding *para*-substituted variants, we note quite remarkable field changes upon Pr formation. This field increase upon transition to Pr can readily be understood in terms of the charge separation that results from the concomitant proton transfer from propC to His278. In addition, in Y165pCNF, we note a strong increase of the local field following the primary photoprocess. Obviously, the putative rearrangement of the H-bond network after photoisomerization alters not only the strength of H-bonding but also the local electric field. It is important to note that no correlation was found between the enol–keto tautomeric equilibrium in Pr and the local electric field. Additionally, the changes in the electric field and the differences observed between the four variants cannot be correlated with the subtle structural alterations in the CBP caused by CNF substitutions. This conclusion also applies to the H-bonding effects (*vide supra*).

Altogether, the present analysis reveals that the local field changes due to noncovalent interactions are smaller than originally expected on the basis of the nitrile stretching frequencies. The results demonstrate that the proton transfer from propC to His278 upon the transition from Meta-F to Pr is associated with large field changes that are sensed by a VSE probe along this reaction pathway (Y165pCNF). The effect is also detectable albeit much weaker at position 192, possibly due to the larger distance from the proton transfer partners. Accordingly, we do not know whether or not the electric field changes that originate in the CBP are propagated to the tongue segment undergoing an α-helix → β-sheet transition even though the latter is known to be linked to the proton transfer. Hence, the present analysis neither confirms nor contradicts the hypothesis of an electric-field-induced restructuring of the tongue segment that is implicated in communicating the chromophore photoisomerization into the activation of the enzymatic output module.

## Conclusions

We have presented an approach to disentangle noncovalent and H-bonding electric field effects of nitrile probes in proteins. It is based on the combined use of nitrile frequencies and intensities as spectroscopic observables and a reliable structural model. This model serves as the input for QMMM calculations to determine reference values for the electric field projected onto the nitrile group and the transition dipole moment (TDM) of its stretching mode. The approach is well-suited for analyzing the electric field contributions in large proteins and monitoring the respective changes along a reaction pathway. A critical evaluation of the accuracy of our approach reveals an overall error of around 25%. This error is dependent on the quality of the reference structural model and the accuracy of the theoretical method.

The application of the present approach to the phytochrome Agp2 demonstrated that the noncovalent electric field effects are smaller than one might have expected in view of earlier experimental findings.^23^ Previous observations highlighted the key importance of intramolecular charge separation (proton transfer) and, in particular, the charge at propC for the secondary structure transition of the tongue. This inspired the hypothesis that the electric field changes might communicate the structural changes in the chromophore binding domain to the tongue.^20,22^ The present results show that the position and orientation of the nitrile probe are critical for the sensed electric field, as documented by comparing the ortho- and para-variants at the same position or the ortho- (para-) variants at different positions. This finding points to a highly heterogeneous distribution of the electric field inside the protein, and even a major change of the electrostatics, i.e., the charge separation between propC and His278 due to the internal proton transfer, is only moderately reflected by the electric field changes at nitrile groups except for the Y165pCNF variant. Therefore, the current study cannot definitively verify the above hypothesis but may help develop an integral theoretical–experimental concept to solve this complex issue. The most challenging task would be to predict “pathways” for a possible electrostatic communication between the chromophore and the tongue, which, however, requires excellent structural models for both the Pfr and Pr state. Then, Stark reporter groups should be inserted at selected points along the predicted routes in a noninvasive way and analyzed spectroscopically on the basis of the present approach.

The second important conclusion drawn from the studies on phytochrome Agp2 refers to changes of H-bonding interactions. We have demonstrated that the strength of H-bonds and their variations can be determined using such nitrile probes. This potential can be utilized to monitor the H-bonding of specific Tyr residues when substituted by para-CNF.

We finally want to emphasize that the present approach may be applied to analyze the reactions of other proteins with respect to the underlying electric field and H-bond changes.

## Supporting Information Available

Additional figures and tables including spectroscopic, calculated, and structural data (PDF)

## ABBREVIATIONS

CBP: chromophore binding pocket
FL: full length
PCM: photosensory core module
RR: resonance Raman
TDM: transition dipole moment
VSE: vibrational Stark effect
WT: wild type
