# Supplementary material for: Hydrogen Bonding and Noncovalent Electric Field Effects in the Photoconversion of a Phytochrome
Source: J Phys Chem B. 2024 Nov 19;128(47):11644–57. doi: 10.1021/acs.jpcb.4c06419 (PMC11613453; doi:10.1021/acs.jpcb.4c06419)
Supplement: Supplementary file 1 — jp4c06419_si_001.pdf [file jp4c06419_si_001.pdf]

Supporting Information

Hydrogen Bonding and Noncovalent Electric Field Effects in the Photoconversion of a Phytochrome

Anh Duc Nguyen[a§], Norbert Michael[b§], Luisa Sauthof[c§], Johannes von Sass[b], Tu Oanh Hoang[a], Andrea Schmidt[c], Mariafrancesca La Greca, Ramona Schlesinger[d], Nediljko Budisa[e], Patrick Scheerer[c*], Maria Andrea Mroginski[a*], Anastasia Kraskov[b*], Peter Hildebrandt[b*]

[*] Correspondence authors

[§] Equal contribution

[a] Technische Universität Berlin, Institut für Chemie, Sekr. C7, Straße des 17. Juni 115, D-10623 Berlin, Germany

[b] Technische Universität Berlin, Institut für Chemie, Sekr. PC14, Straße des 17. Juni 135, D-10623 Berlin, Germany

[c] Charité − Universitätsmedizin Berlin, corporate member of Freie Universität Berlin and Humboldt-Universität zu Berlin, Institute of Medical Physics and Biophysics, Group Structural Biology of Cellular Signaling, Charitéplatz 1, D-10117 Berlin, Germany

[d] Freie Universität Berlin, Experimental Physics: Genetic Biophysics, Arnimallee 14, D-14195 Berlin, Germany

[e] Department of Chemistry, University of Manitoba, 144 Dysart Rd, R3T 2N2 Winnipeg, Manitoba, Canada

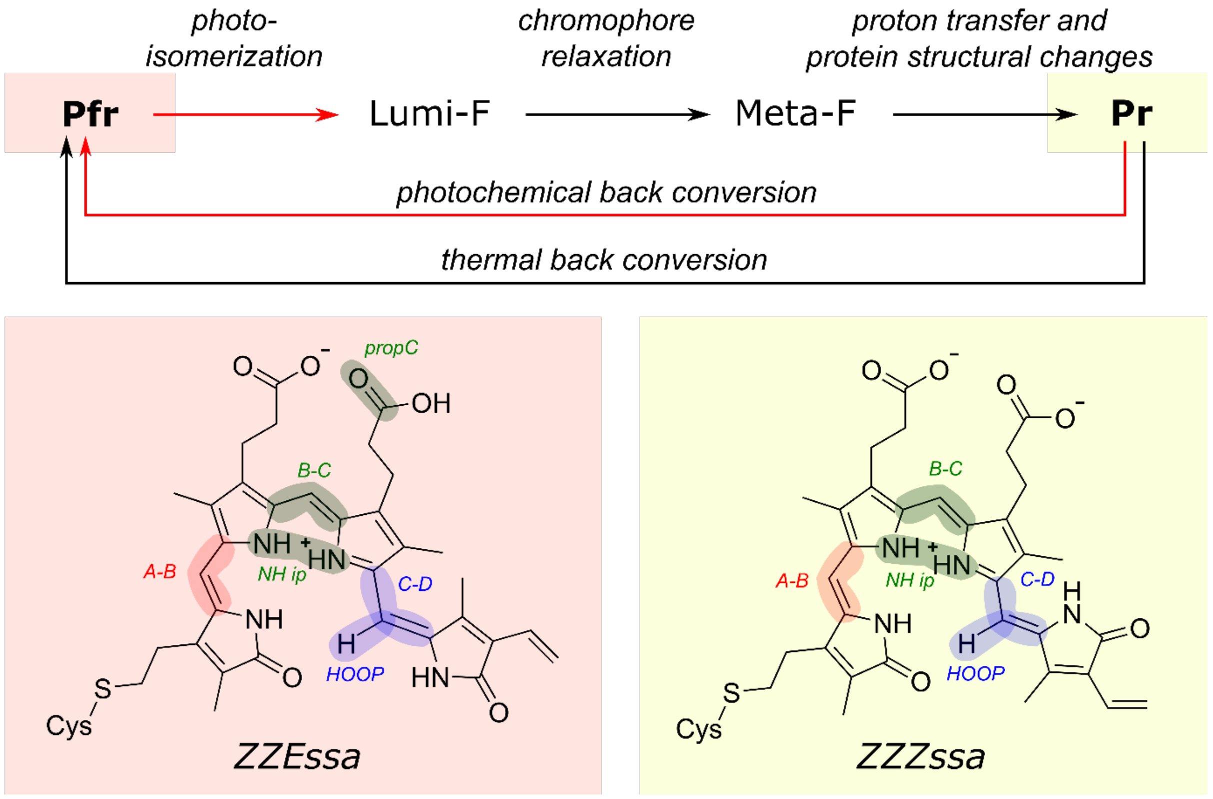

Fig. S1. The Pfr → Pr photocycle of Agp2 and the corresponding biliverdin structures. Photochemical and thermal reaction steps are represented by red and black arrows. The ZZEssa and ZZZssa chromophore structures are highlighted in light red and yellow, respectively. Localization of Raman modes as listed in Figure 2 are illustrated in the structural formulas in red (A-B), green (B-C), and blue (C-D).

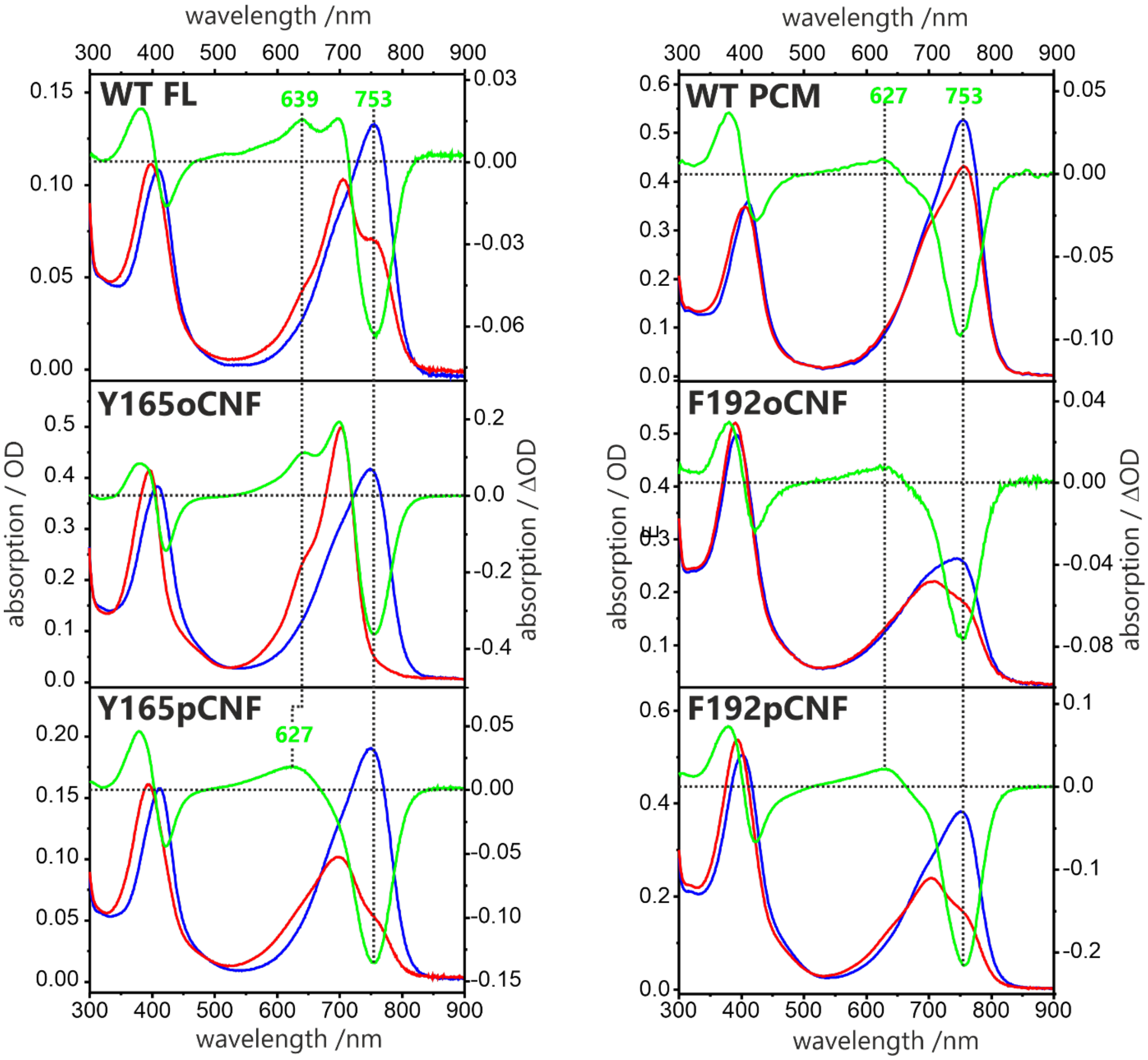

Fig. S2. UV-vis absorption spectra of Agp2 and its photosensory core module (PCM) variants including pCNF and oCNF residues. The blue and red spectra refer to the Pfr and Pr state, respectively. Green traces indicate the “Pr minus Pfr” difference spectra, characterized by the dotted horizontal zero-line. Spectra of the full-length (FL) and PCM Agp2 as well as of the pCNF variants were taken from our previous work.[1,2] The green labels mark the negative and positive peaks of the difference spectra, corresponding to Pfr and Pr, respectively. Among all proteins, the Pfr peaks are the same at 753 ± 2 nm, the peaks of Pr with predominant keto tautomer at 639 ± 2 nm, and those with predominant enol tautomer 627 ± 2 nm. Dark reversion is slower for all variants as compared to the WT proteins.

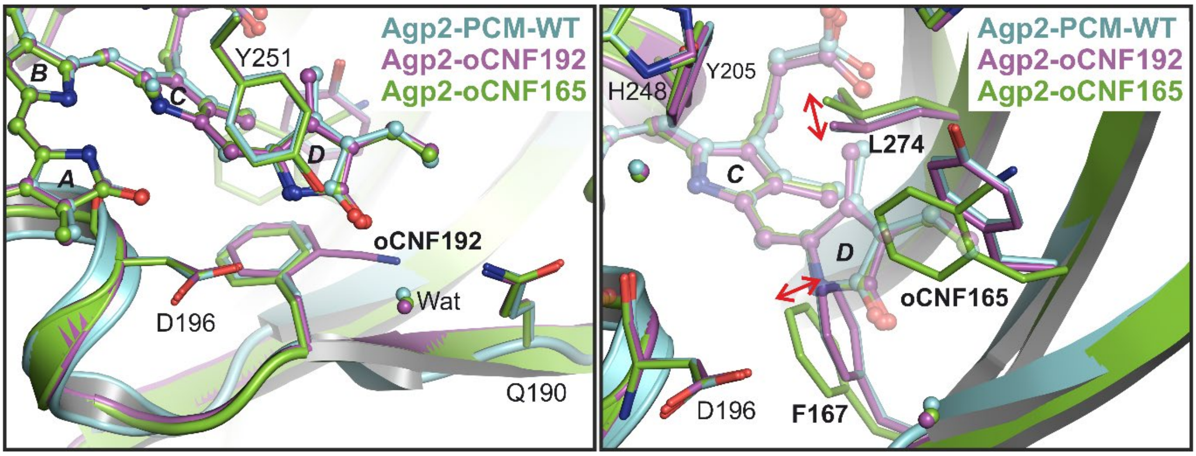

Fig. S3. Close-up view of the chromophore binding pocket with the oCNF residues of F192oCNF (magenta, PDB entry 9G8D), Y165oCNF (green, PDB entry 9G8C), compared to the WT Agp2-PCM (cyan, PDB entry 6G1Y) crystal structures in the Pfr states. **Left**, crystal structure of F192oCNF, which is in very good agreement with the superimposed structure of WT Agp2-PCM. **Right**, crystal structure of Y165oCNF, which shows a few differences compared to WT Agp2-PCM. The residues Phe167 and Leu274 close to Y165oCNF are shifted by 0.9 Å and 0.8 Å, respectively. The protein backbone, chromophore, and selected residues are depicted as cartoon, sticks/balls and sticks, respectively.

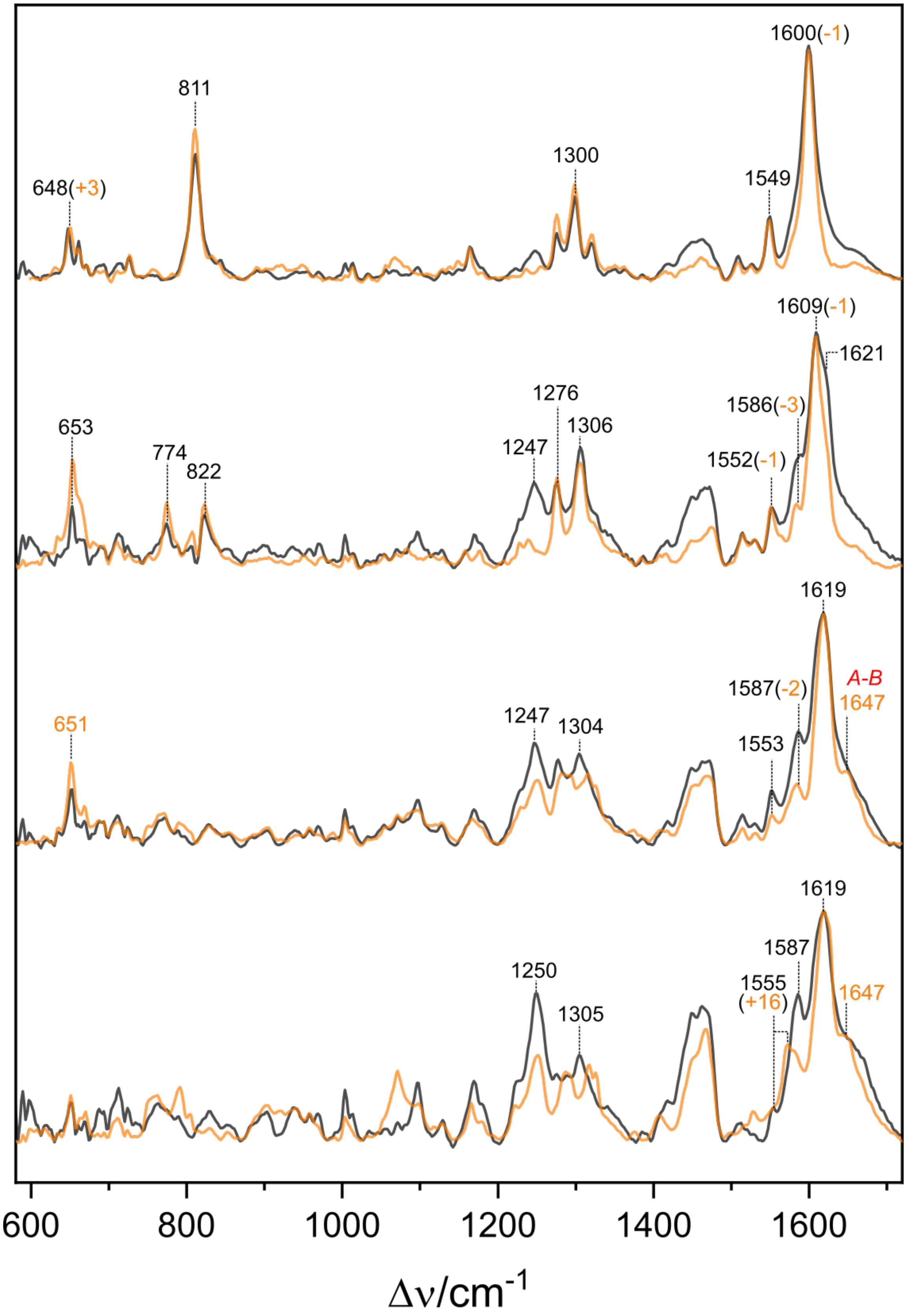

Fig. S4. RR spectra of Agp2-F192oCNF (black) in the various states of the Pfr → Pr conversion, compared to the WT Agp2-FL (orange). From top to bottom: Pfr, Lumi-F, Meta-F, Pr. Spectra of WT Agp2-FL were taken from previous work.[1–3]

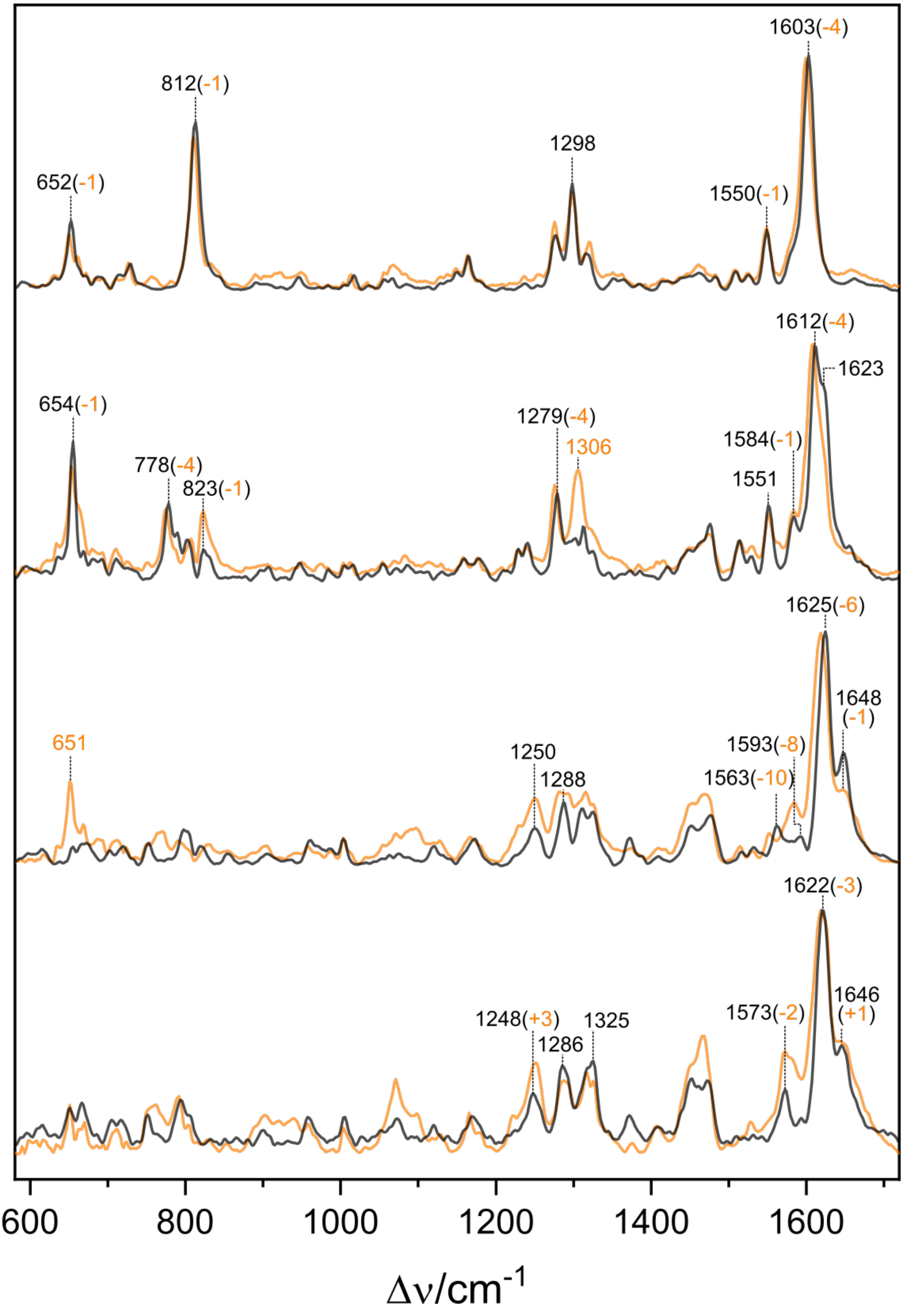

Fig. S5. RR spectra of Agp2-Y165oCNF (black) in the various states of the Pfr → Pr conversion, compared to the WT Agp2-FL (orange). From top to bottom: Pfr, Lumi-F, Meta-F, Pr. Spectra of WT Agp2-FL were taken from previous work.[1–3]

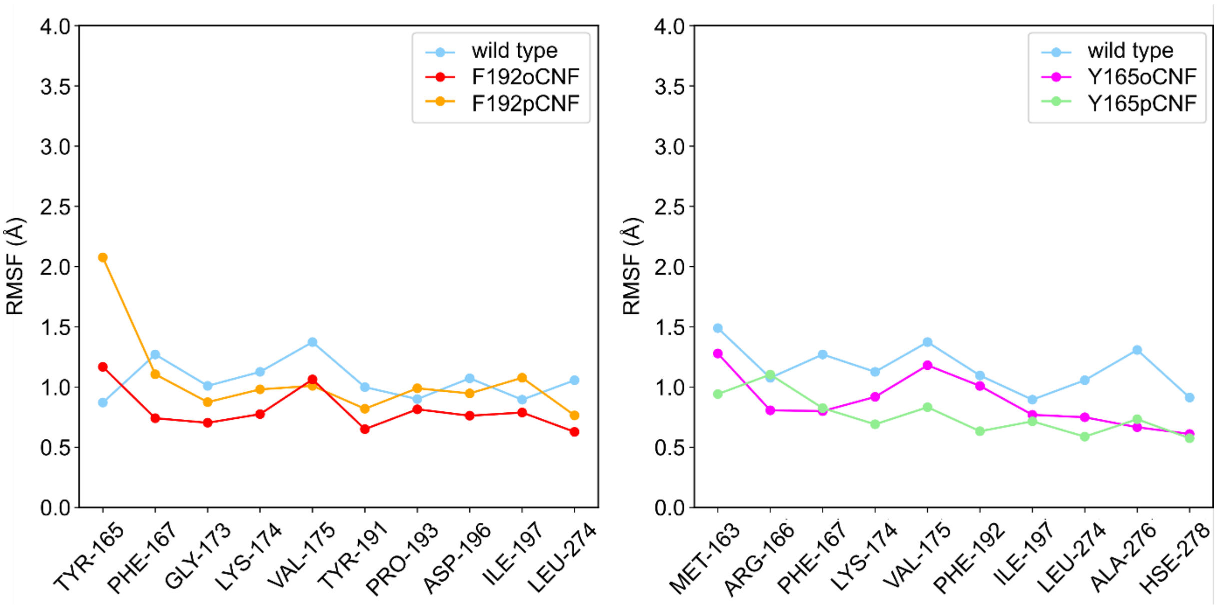

Fig. S6. Comparison between the average RMSF values of the WT protein (blue) and the Agp2-PCM variants with a cyanophenylalanine at position 192 (left panel) and 165 (right panel) for residues in the immediate environment of the reporter group. This analysis is conducted over the course of the last 30 ns of the MD simulation.

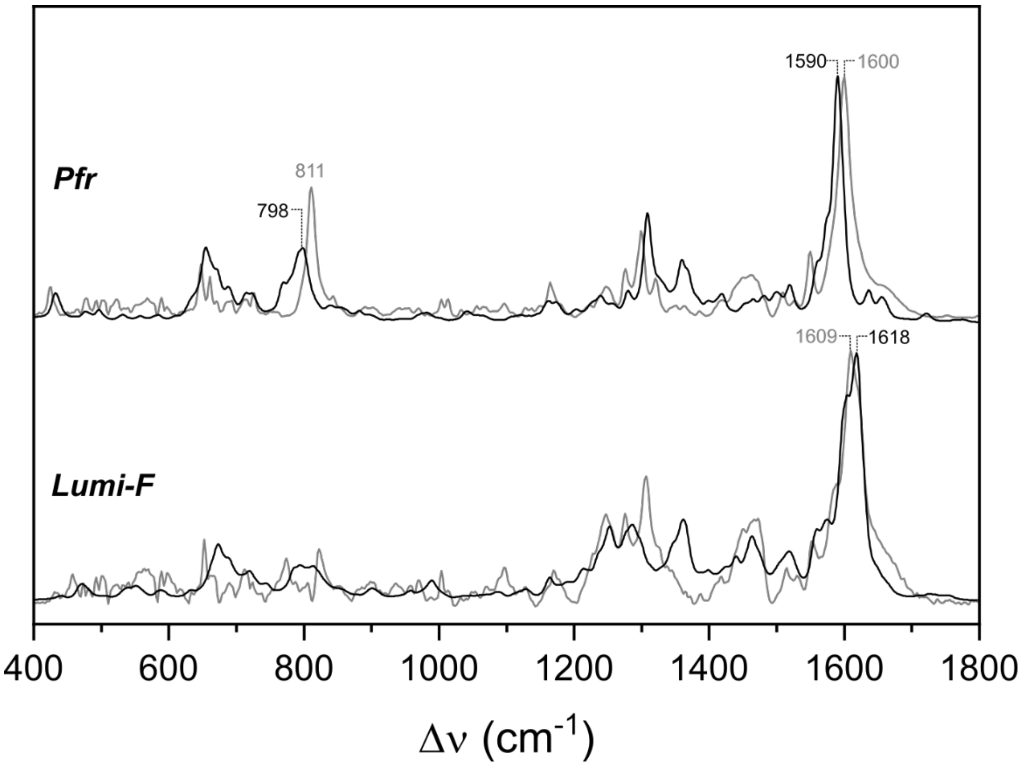

Fig. S7-1. Calculated (black) and experimental (grey) RR spectra of Agp2-F192oCNF in the Pfr and Lumi-F state. The calculated spectra represent the average of 50 QMMM-snapshots.

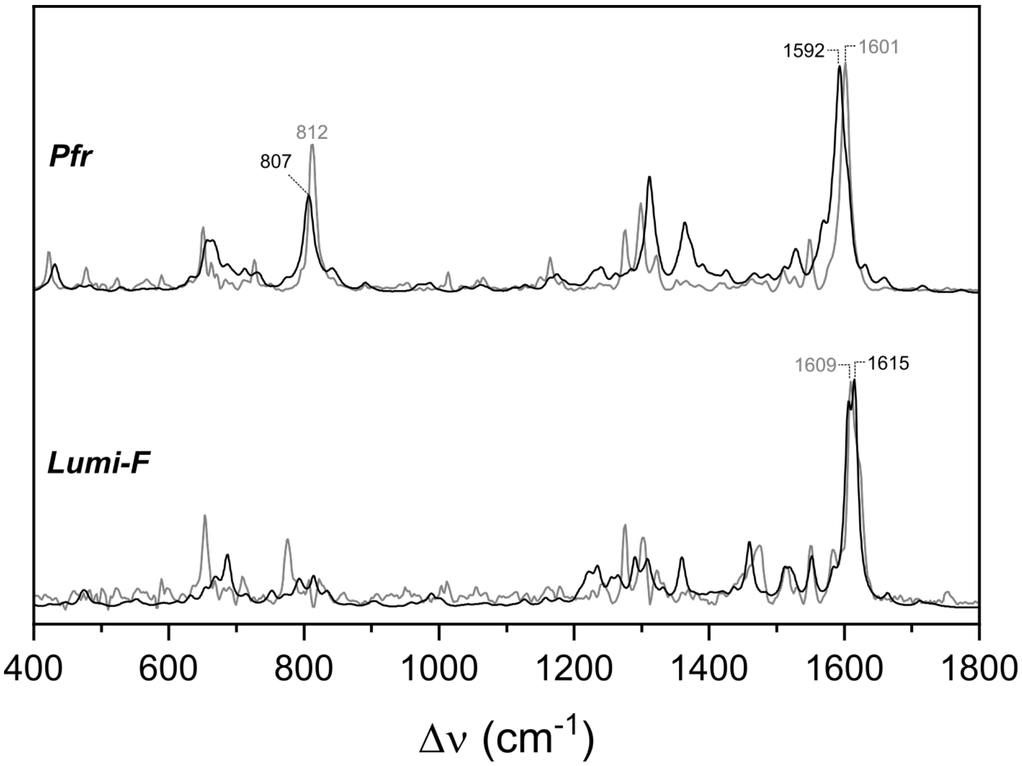

Fig. S7-2. Calculated (black) and experimental (grey) RR spectra of Agp2-F192pCNF in the Pfr and Lumi-F state. The calculated spectra represent the average of 50 QMMM-snapshots. Spectra of Agp2-F192pCNF were taken from previous work.[1]

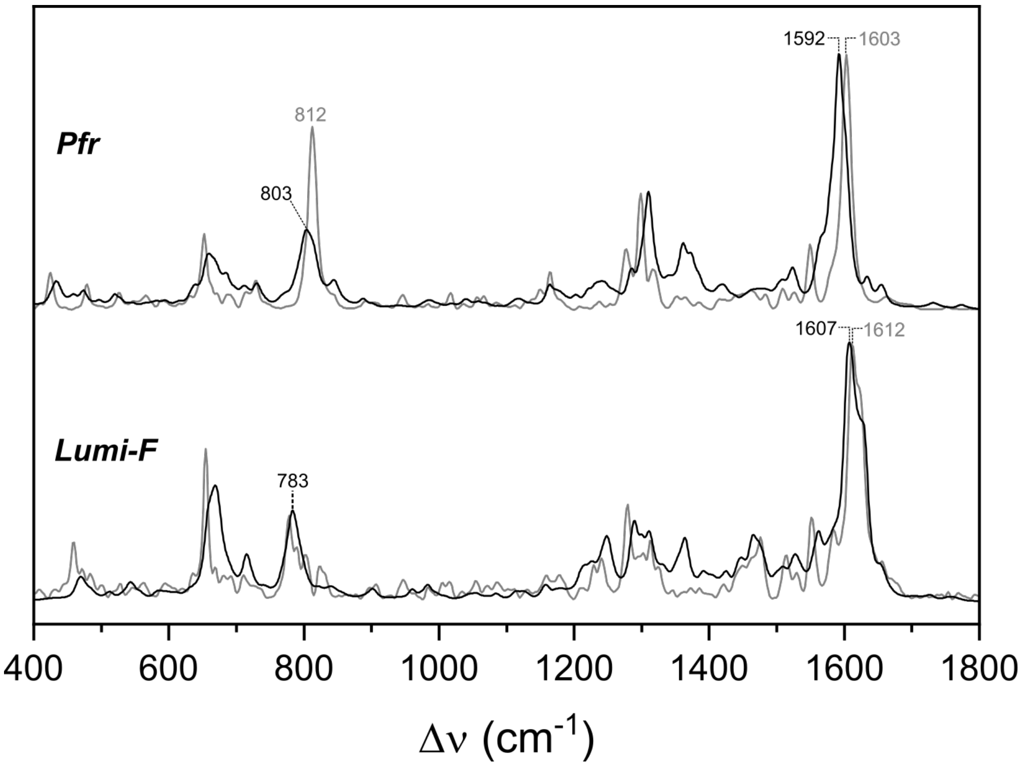

Fig. S7-3. Calculated (black) and experimental (grey) RR spectra of Agp2-Y165oCNF in the Pfr and Lumi-F state. The calculated spectra represent the average of 50 QMMM-snapshots.

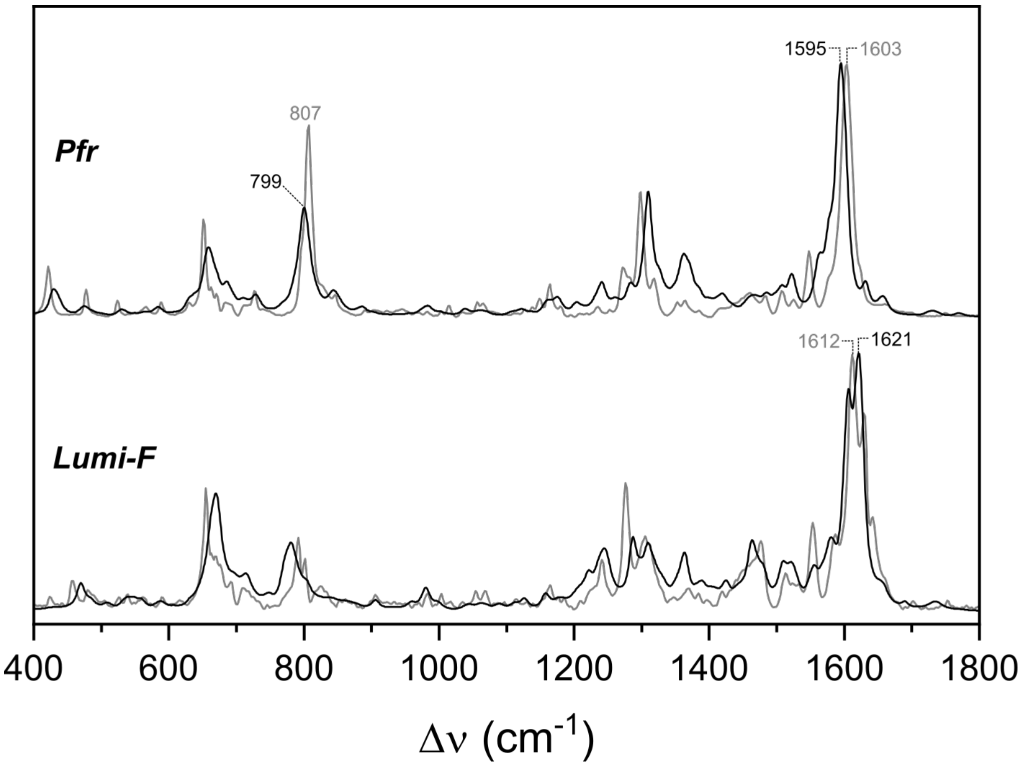

Fig. S7-4. Calculated (black) and experimental (grey) RR spectra of Agp2-Y165pCNF in the Pfr and Lumi-F state. The calculated spectra represent the average of 50 QMMM-snapshots. Spectra of Agp2-Y165pCNF were taken from previous work.[1]

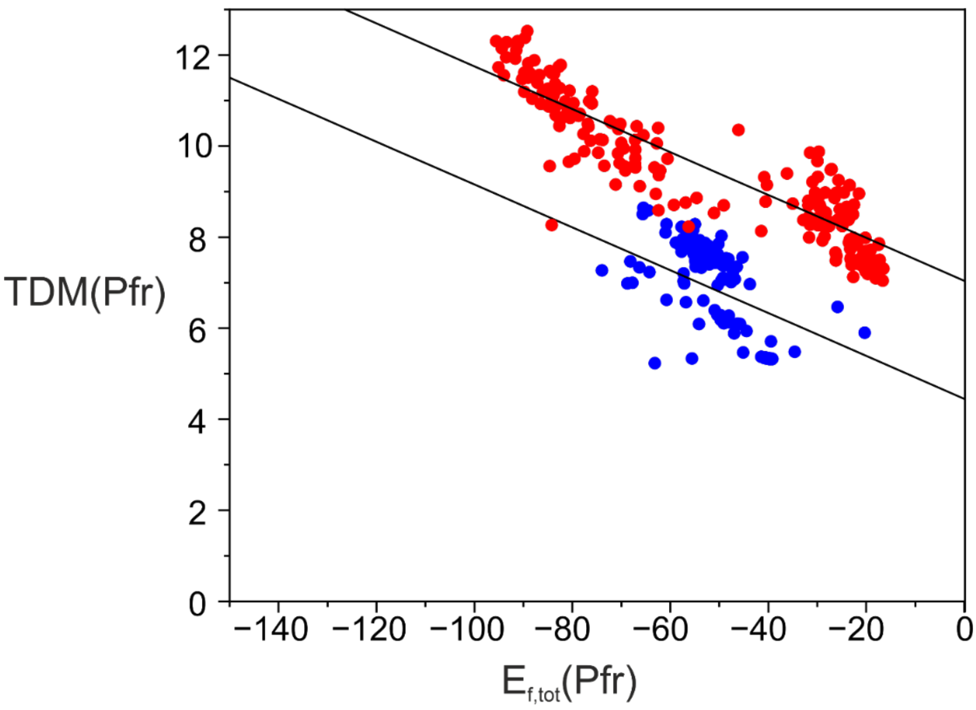

Fig. S8. Plot of the calculated transition dipole moments versus the electric field for all Pfr QM/MM snapshots. Data of *ortho*-CNF- and *para*-CNF-variants are shown in blue and red, respectively. The linear fits to the data according to eq. 4 yielded the same slope of $-0.047\sqrt{\frac{km}{mol}} \cdot cm/MV$ in both cases but different intercepts of 4.44 and 7.04 $(km \cdot Mol)^{1/2}$ for the *ortho*-CNF and *para*-CNF species, respectively.

Table S1. Data collection and refinement statistics of the Stark label variants F192oCNF and Y165oCNF.

	**F192oCNF**[a] (PDB entry 9G8D)	**Y165oCNF**[a] (PDB entry 9G8C)
**Data collection** (wavelength)	ESRF, ID30B<br>λ = 0.97625 Å	ESRF, ID23-2<br>λ = 0.87317 Å
Space group	$P2_12_12_1$	$P2_12_12_1$
Cell dimensions		
a, *b*, *c* (Å)	74.64, 93.58, 173.95	74.72, 93.76, 174.57
α, β, γ (°)	90.0, 90.0, 90.0	90.0, 90.0, 90.0
Resolution (Å)	45.83 – 2.06<br>(2.11 – 2.06)[b]	45.95 – 1.90<br>(1.95 – 1.90)[b]
$<I/\sigma(I)>$<br>*cc1/2*	6.0 (1.5)<br>85.7 (20.1)	11.4 (1.5)<br>99.8 (73.2)
Completeness (%)	99.9 (99.5)	99.7 (97.8)
Multiplicity	6.5 (5.3)	12.8 (10.6)
**Refinement**		
No. Reflections	75,997	96,932
R_{cryst} / R_{free} (%)	18.7 / 22.6	18.5 / 22.3
No. atoms<br>Protein<br>Ligand/ion<br>Water	 7,550<br>144<br>656	 7,618<br>191<br>958
B-factors		
Protein<br>Ligand/ion<br>Water	35.6<br>31.8<br>42.7	26.4<br>29.9<br>35.9
RMSD[c]		
Bond lengths (Å)	0.013	0.014
Bond angles (°)	1.63	1.69
Ramachandran plot[d]		
Favoured (%)	98.9	98.8
Allowed (%)<br>Outlier (%)	1.1<br>0.0	1.2<br>0.0

[a] One crystal was used; [b] highest resolution shell is shown in parenthesis; [c] RMSD, root mean square deviations; [d] Ramachandran plot calculated by MolProbity[4]

Table S2. Nitrile stretching frequencies[a].

	Observed frequencies					Normalized frequencies at RT			
variant	Peak	Pfr	Lumi-F	Meta-F	Pr	Pfr	Lumi-F	Meta-F	Pr
T/K[b]		140	140	240	300	300	300	300	300
Y165oCNF	P1	2231.5 (2231.5)	2232.7 (2231.5)	2225.1 (2225.6)	2226.9 (2225.9)	2225.9	2227.1	2225.4	2226.9
	P2	2237.4 (2237.4)	2237.7 (2237.4)	2228.9 (2229.9)	2231.0 (2230.4)	2230.4	2230.7	2229.4	2231.0
F192oCNF	P1	2228.2 (2228.2)	2228.2 (2228.2)	2226.9 (2226.6)	2226.3 (2226.6)	2226.6	2226.6	2226.9	2226.3
	P2	2236.8 (2236.8)	2236.7 (2236.8)	-	2231.5 (2231.8)	2231.8	2231.7	-	2231.5
Y165pCNF	P1	-	-	-	-	-	-	-	-
	P2	2240.3 (2240.3)	2237.6 (2240.3)	2239.4 (2238.9)	2232.4 (2237.4)	2237.4	2234.7	2237.9	2232.4
	P3	-	-	-	2241.6 (2237.4)	-	-	-	2241.6
F192pCNF	P1	2226.5 (2226.5)	2226.9 (2226.5)	2226.5 (2226.3)	2225.5 (2224.9)	2224.9	2225.3	2225.1	2225.5
	P2	2233.3 (2233.2)	2233.5 (2233.2)	2232.0 (2231.7)	2229.0 (2230.5)	2230.5	2230.8	2230.8	2229.0

[a] Frequencies are given in cm^{-1}.
[b] Values in parenthesis refer to the spectra of Pfr measured at the same temperature.

Table S3. Integral intensities and their normalized values.

variant	peak	Measured integral intensities $I(i)$						Normalized intensities $I(i)$			
		Pfr 140 K	Lumi-F 140 K	Pfr 240 K	Meta-F 240 K	Pfr 300 K	Pr 300 K	Pfr 300 K	Lumi-F 300 K	Meta-F 300 K	Pr 300 K
Y165oCNF	total	0.00392	0.00384	0.00287	0.00264	0.00764	0.00763	7.64	7.484	7.028	7.63
	P1	0.0008	0.00094	0.00179	0.00165	0.00111	0.00372	1.11	1.832	4.392	3.72
	P2	0.00312	0.0029	0.00108	0.00099	0.00653	0.00391	6.53	5.652	2.635	3.91
F192oCNF	total	0.01691	0.0167	0.00624[a]	0.00443[a]	0.01286	0.01404	12.86	12.70	9.130[a]	14.04
	P1	0.011	0.0115	-	-	0.00922	0.00861	9.22	8.746		8.61
	P2	0.00591	0.0052	-	-	0.00364	0.00543	3.64	3.955		5.43
Y165pCNF	total	0.00467	0.01091	0.00321	0.00235	0.02642	0.0378	2.642	6.172	1.934	3.780
	P1	-	-	-	-	-	-	-	-	-	-
	P2	-	-	-	-	-	0.02094	0.731	-	-	2.094
	P3	-	-	-	-	-	0.01686	8.134	-	-	1.686
F192pCNF	total	0.02704	0.02401	0.00919	0.00929	0.01516	0.01796	1.516	1.346	1.532	1.796
	P1	0.00869	0.0098	0.00241	0.00353	0.00352	0.00268	0.352	0.549	0.582	0.268
	P2	0.01835	0.01421	0.00678	0.00576	0.01164	0.01528	1.164	0.797	0.950	1.528

Table S4. Calculated total electric fields for the Pfr states by QMMM and the corresponding quantities for the intermediates determined via Eq. 5. Calculated fields for the Lumi-F model (by QMMM) are given in parenthesis.

	$E_{F,tot,Pfr}$ calc. (MV/cm)	$E_{F,tot,Lumi}$ (MV/cm)	$E_{F,tot,Meta}$ (MV/cm)	$E_{F,tot,Pr}$ (MV/cm)
Y165oCNF	–53.41	–52.13 (–42.61)	–48.30	–53.33
F192oCNF	–45.4	–44.39 (–86.93)	–20.17[a]	–52.59
Y165pCNF	–73.84	–221.70 (–83.57)	–33.44	–128.72
F192pCNF	–28.76	–22.56 (–19.09)	–29.50	–41.32

References

(1) Kraskov, A.; Von Sass, J.; Nguyen, A. D.; Hoang, T. O.; Buhrke, D.; Katz, S.; Michael, N.; Kozuch, J.; Zebger, I.; Siebert, F. et al. Local Electric Field Changes during the Photoconversion of the Bathy Phytochrome Agp2. *Biochemistry* **2021**, *60* (40), 2967–2977. https://doi.org/10.1021/acs.biochem.1c00426.

(2) Kraskov, A.; Nguyen, A. D.; Goerling, J.; Buhrke, D.; Velazquez Escobar, F.; Fernandez Lopez, M.; Michael, N.; Sauthof, L.; Schmidt, A.; Piwowarski, P. et al. Intramolecular Proton Transfer Controls Protein Structural Changes in Phytochrome. *Biochemistry* **2020**, *59* (9), 1023–1037. https://doi.org/10.1021/acs.biochem.0c00053.

(3) Velazquez Escobar, F.; Piwowarski, P.; Salewski, J.; Michael, N.; Fernandez Lopez, M.; Rupp, A.; Qureshi, M. B.; Scheerer, P.; Bartl, F.; Frankenberg-Dinkel, N. et al. A Protonation-Coupled Feedback Mechanism Controls the Signalling Process in Bathy Phytochromes. *Nat. Chem.* **2015**, *7* (5), 423–430. https://doi.org/10.1038/nchem.2225.

(4) Davis, I. W.; Leaver-Fay, A.; Chen, V. B.; Block, J. N.; Kapral, G. J.; Wang, X.; Murray, L. W.; Arendall, W. B.; Snoeyink, J.; Richardson, J. S. et al. MolProbity: All-Atom Contacts and Structure Validation for Proteins and Nucleic Acids. *Nucleic Acids Res.* **2007**, *35* (SUPPL.2), 375–383. https://doi.org/10.1093/nar/gkm216.
